# Amphipathic dendritic poly-peptides carrier to deliver antisense oligonucleotides against multi-drug resistant bacteria in vitro and in vivo

**DOI:** 10.1186/s12951-022-01384-y

**Published:** 2022-04-02

**Authors:** Zhou Chen, Yue Hu, Xinggang Mao, Dan Nie, Hui Zhao, Zheng Hou, Mingkai Li, Jingru Meng, Xiaoxing Luo, Xiaoyan Xue

**Affiliations:** 1 Department of Pharmacology, School of Pharmacy, Fourth Military Medical University, Xi’an, 710032 Shaanxi China; 2 Department of Neurosurgery of Xijing Hospital, Fourth Military Medical University, Xi’an, 710032 Shaanxi China

**Keywords:** Antibacterial strategy, Antisense, Dendritic poly-peptides, Multidrug-resistant bacteria, Nanoparticles, Delivery, Oligonucleotide

## Abstract

**Background:**

Outbreaks of infection due to multidrug-resistant (MDR) bacteria, especially Gram-negative bacteria, have become a global health issue in both hospitals and communities. Antisense oligonucleotides (ASOs) based therapeutics hold a great promise for treating infections caused by MDR bacteria. However, ASOs therapeutics are strangled because of its low cell penetration efficiency caused by the high molecular weight and hydrophilicity.

**Results:**

Here, we designed a series of dendritic poly-peptides (DPP1 to DPP12) to encapsulate ASOs to form DSPE-mPEG2000 decorated ASOs/DPP nanoparticles (DP-AD1 to DP-AD12) and observed that amphipathic DP-AD2, 3, 7 or 8 with a positive charge ≥ 8 showed great efficiency to deliver ASOs into bacteria, but only the two histidine residues contained DP-AD7 and DP-AD8 significantly inhibited the bacterial growth and the targeted gene expression of tested bacteria in vitro*.* DP-AD7_anti-*acpP*_ remarkably increased the survival rate of septic mice infected by ESBLs-*E. coli,* exhibiting strong antibacterial effects in vivo.

**Conclusions:**

For the first time, we designed DPP as a potent carrier to deliver ASOs for combating MDR bacteria and demonstrated the essential features, namely, amphipathicity, 8–10 positive charges, and 2 histidine residues, that are required for efficient DPP based delivery, and provide a novel approach for the development and research of the antisense antibacterial strategy.

**Graphical Abstract:**

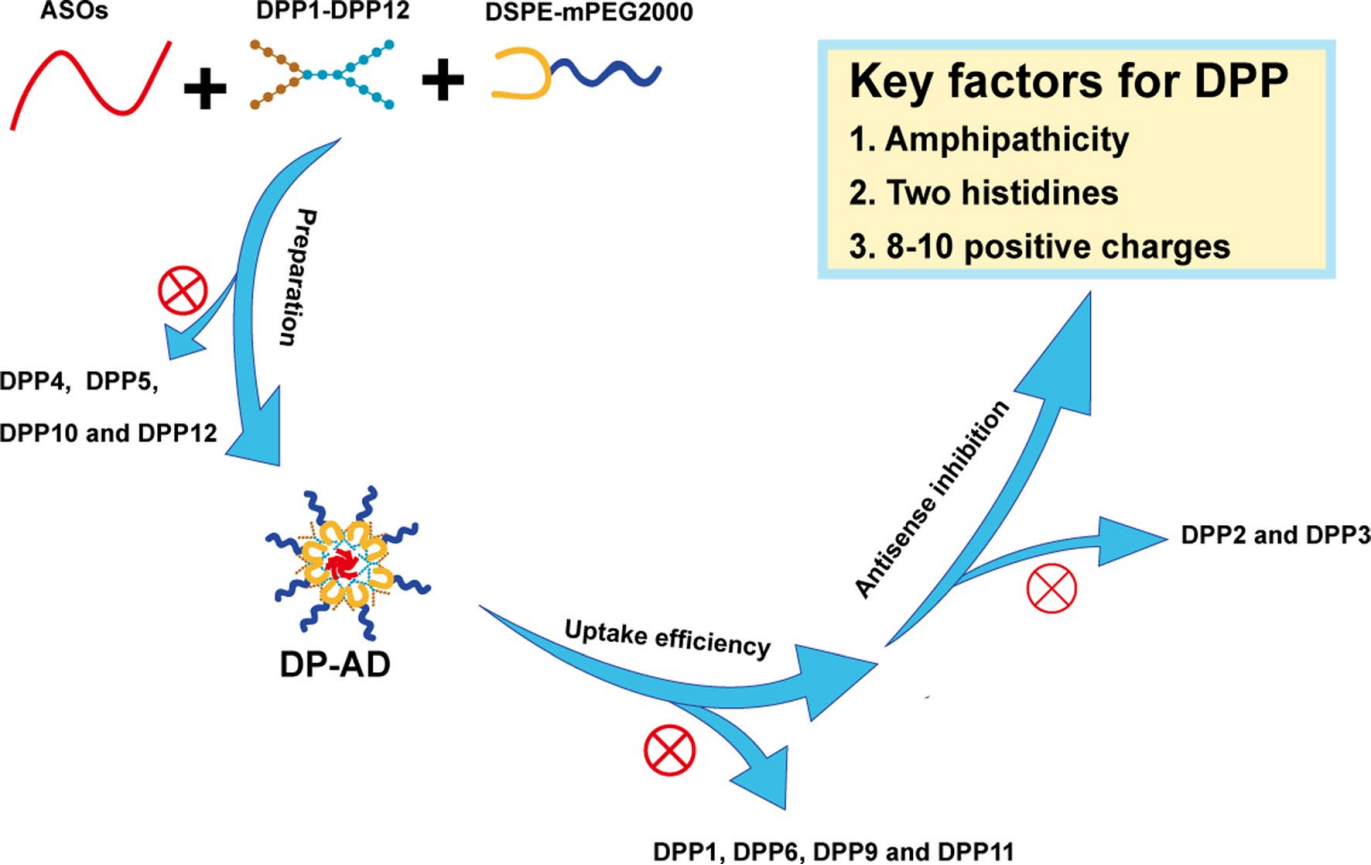

**Supplementary Information:**

The online version contains supplementary material available at 10.1186/s12951-022-01384-y.

## Background

Nowadays, multidrug-resistant (MDR) bacterial infections are becoming difficult to cure, especially those caused by the organisms on the World Health Organization priority bacteria list [1–4]. So it is imperative to search for and develop novel strategies to fight against MDR bacteria. With the advantages of high specificity, easy design and synthesis, antisense oligonucleotides (ASOs) based antibacterial technology has emerged as a promising approach to reverse the resistance or inhibit the growth of bacteria by blocking the expression of critical genes [5]. Thus far, numerous functional genes have been validated as potential antisense antibacterial targets, such as *vanA* (encoding the glycopeptide-resistant related protein) and *acpP* (encoding the survival-essential acyl carrier protein involved in fatty acid biosynthesis) [6–8]. Our laboratory also identified several genes, such as *mecA*, *acrB* and *rpoD* (encoding the survival essential RNA polymerase primary δ^70^)*,* that can be targeted by different types of ASOs. These ASOs can inhibit the expression of targeted genes and ultimately abolish the antibiotic resistance of bacteria or kill bacteria in vitro and in vivo [9–12].

Despite the advantages of ASOs based antibacterial agents and significant technological advancement in oligonucleotide chemical modification, free ASOs without any vector can hardly penetrate the cellular membrane because of their high molecular weight and hydrophilicity. Furthermore, the bacterial cell wall presents another obstacle to ASOs delivery, especially the outer-membrane lipopolysaccharide layer in Gram-negative bacteria, which blocks the entrance of antibiotics and other foreign compounds [13, 14]. Therefore, lacking delivery system seriously hampered antibacterial ASOs clinical application.

Two common strategies were developed for ASOs delivery. One involves the encapsulation of ASOs into nanoparticles (NPs) by using cationic materials, such as lipofectamine 2000 (LF2000), bolaamphiphiles and green tea catechin [11, 14–17], However, thus far, little is known about why these NPs showed a considerably lower delivery efficacy and higher cytotoxicity in bacteria than in mammalians [18, 19]. Another more widely studied strategy is the conjugation of ASOs with different cell penetrating peptides (CPPs) covalently to generate a new compound ASOs-CPP [9, 10]. However, even with the currently most effective CPP, RXRRXRRXRRXRXB (X is 6-aminohexanoic acid and B is β-alanine), a high dosage of ASOs-CPP is still required for satisfactory therapeutic effect, probably due to the low drug loading capacity of covalent CPPs. In addition, large-scale synthesis and purification of ASOs-CPP products are time-consuming and expensive [18–20].

An alternative strategy used in mammalian cells is to complex ASOs with CPPs non-covalently to form NPs, which exhibited high ASOs loading capacity, low cytotoxicity and low immunogenicity [21]. However, those CPPs/ASOs NPs also showed lower transfection efficiency in bacteria. For example, CADY peptide NPs could deliver ASOs molecular into mammalian cells more efficiently than in bacteria [22]. We assumed that this may be related to the special structure of bacteria, and the successful design of ASOs/peptide NPs for bacteria may require systematic research on the peptide structures and sequences. Various factors, including the geometry structure of peptides, hydrophobicity, Histidine (His) and the number of positive charges, play essential roles in the delivery process in mammalian cells [19, 23, 24]. For example, dendritic peptides hybridized with lipids have higher transfection in mammalian cells compared with their linear counterparts [23], and the melittin peptide with His residues exhibited a higher transfection efficiency than the counterparts without His residues [24].

Here, to systematically study the factors influencing uptake efficiency of peptide NPs in bacteria, we designed a series of dendritic poly-peptides (DPP1–DPP12) with various parameters, including hydrophobicity, the number of positive charges, and DPP amino acid types, to screen DPPs that can efficiently deliver ASOs into bacteria. Then, the antibacterial activity of ASOs targeting *acpP* delivered by DPPs was evaluated in vitro and in vivo. ASOs was encapsulated by DPP to form 1,2-distearoyl-sn-glycero-3-phosphoethanolamine-N-methoxy(polyethylene glycol)-2000 (DSPE-mPEG2000) decorated NPs (DP-AD, where AD stands for ASOs/DPP NPs). We demonstrated that amphipathic DPP2, 3, 7 and 8, which contained 8–10 positive charges, showed a notably higher efficiency than hydrophilic DPP1 and DPP6 to deliver ASOs into bacteria, but only DP-AD7 and DP-AD8 containing two His residues showed better gene knockdown and growth inhibitory effect in tested strains in vitro. Importantly, DP-AD7 targeting *acpP*, significantly increased the survival rate and reduced the bacterial colony forming units (CFU) in the organs of septic mice infected by extended spectrum beta-lactamases producing (ESBLs)-*E. coli*. Conclusively, for the first time, our research clarified the essential factors required for the assembly of DP-AD with the best delivery performance into bacteria, demonstrating that DPP based non-covalent complexion strategy is a promising approach to deliver antisense antibacterial agents into bacteria.

## Results and discussion

### The design of DPPs

**Scheme 1:**
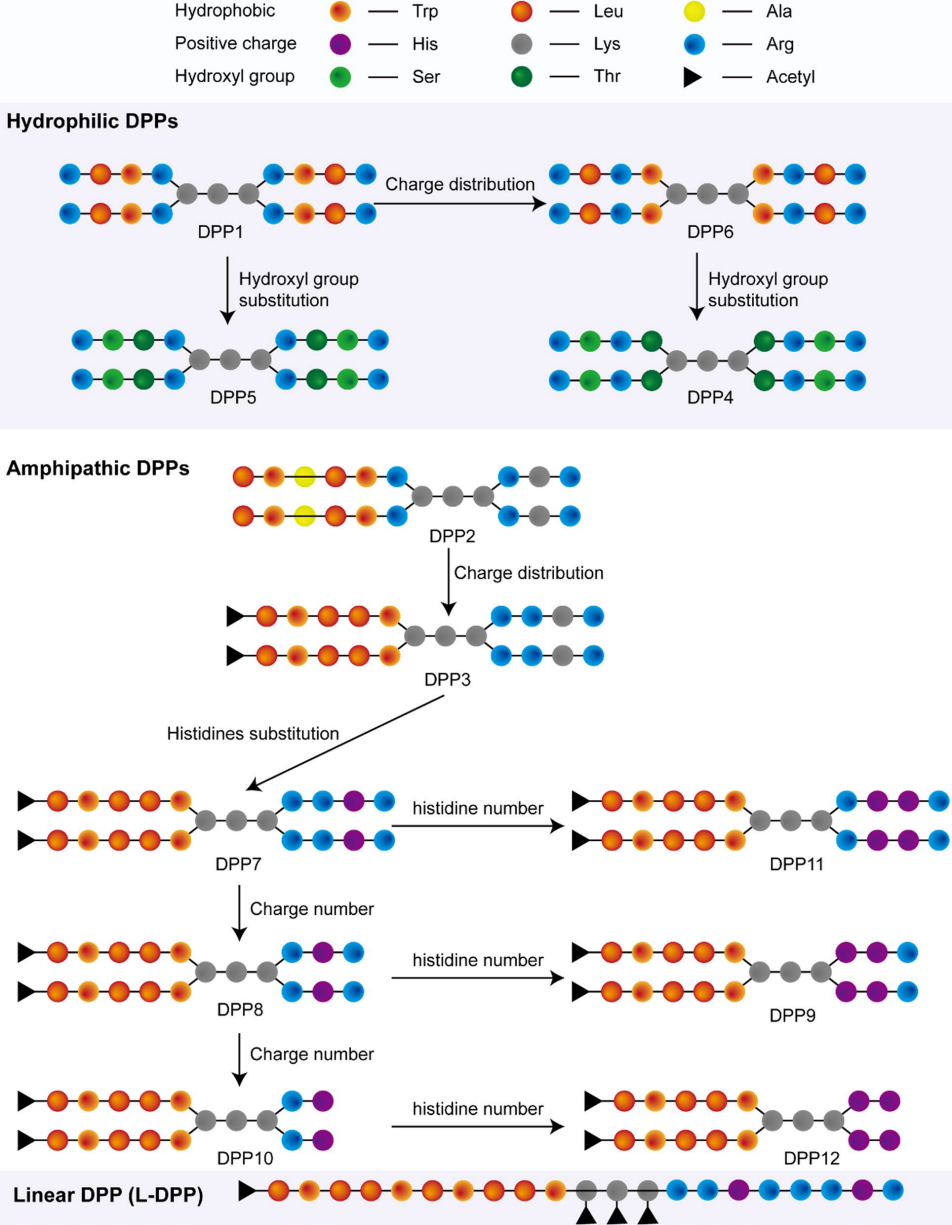
DPP design. The positive charges in hydrophilic DPP1 were averaged to obtain DPP2. Trp and Leu in DPP1 and DPP6 were substituted with hydroxyl containing Ser and Thr to obtain DPP4 and DPP5, respectively. The positive charges in amphipathic DPP2 were aggregated on one side to obtain amphipathic DPP3, and two Lys residues in the positive arms were substituted with two His residues to yield DPP7. The positive charge was reduced to obtain DPP8 and DPP10. The number of His residues in DPP7, 8 and 10 were increased to four to obtain DPP11, DPP9 and DPP12, respectively. In addition, a linear DPP (L-DPP) with the same sequence as DPP7 was also synthesized as controls

To screen the DPPs that can transfect ASOs efficiently into bacteria, we synthesized 12 DPPs with four terminal branches and a linear counterpart by solid-phase peptide synthesis. The DPPs were designed to have different hydrophobicity and positive charge distributions by introducing hydrophobic leucine (Leu), tryptophan (Trp) and alanine (Ala), and hydrophilic cationic arginine (Arg) and lysine (Lys) (Scheme 1, Additional file 1: Fig. S1–S3, Tables S1, S2), which are commonly used to confer cell-penetrating capacities on peptides [25–27]. In detail, DPP1, DPP4, DPP5 and DPP6 were hydrophilic, while DPP2, DPP3 and DPP7–DPP12 were amphipathic. The hydrophobic Leu and Trp in DPP1 and DPP6 were replaced with hydrophilic hydroxyl-containing threonine (Thr) and serine (Ser), respectively, to form the strictly hydrophilic DPP4 and DPP5, given that the hydroxyl group may enhance the hydrophilicity and adhesion between bacteria and NPs [28, 29]. With positive charge distribution as another significant factor determining the delivery efficiency of CPPs [21], different distribution of Arg residues in DPP1 and DPP4, as the primary source of positive charges in DPPs, were used to generate DPP6 and DPP5, respectively, to explore the effects of charge distribution on the delivery efficiency. In addition, the His residues in CPP sequences enhances NPs endosomal escape in mammalian cells [24], thus two Lys residues in DPP3 were replaced by two His residues to generate DPP7 to test whether His could improve DPP transfection efficiency in bacteria. To further clarify the effects of the number of positive charges and His residues in DPP, we designed DPP8–DPP12 based on DPP7 sequence by increasing or decreasing the number of His and Arg residues. The counterpart linear DPP (L-DPP) with the same sequence as DPP7 was also synthesized as the control.

### Preparation and characterization of DP-AD

**Fig. 1:**
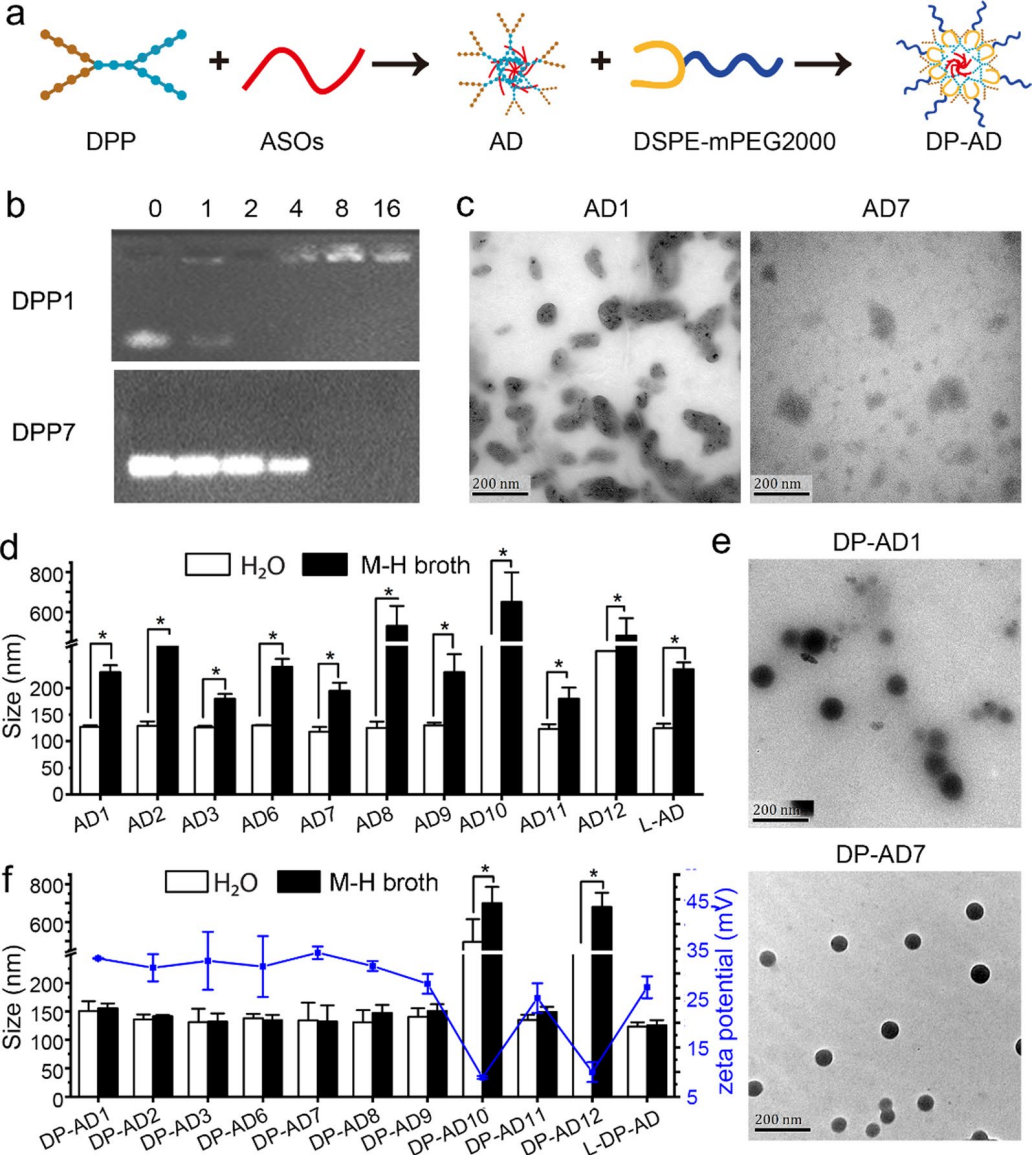
Preparation and characteristics of DP-AD. **a** Schematic of the two-step preparation of DP-AD. **b** Screening of the best N/P molar ratios ranging from 1 to 16 by 1% agarose gel electrophoresis. The upper and lower images indicated hydrophilic DP-AD1 and amphipathic DP-AD7, respectively. **c** TEM images of AD1 and AD7. **d** The size of ADs in dd H_2_O (white columns) and diluted with equal volume of M–H broth (black columns). **e** TEM images of DP-AD1 and DP-AD7. **f** The size of DP-AD in dd H_2_O (white columns) and diluted with equal volume of M–H broth (black columns), and zeta potential (blue line) of DP-AD in dd H_2_O determined by DLS. Bar = 200 nm

DP-AD were prepared using a two-step protocol (Fig. 1a and Additional file 1: Table S3). Briefly, DPP and ASOs were mixed and incubated at 37 °C for 30 min, added with DSPE-mPEG2000, and then incubated for another 30 min. In the present study, all ASOs were synthesized by 2′-OMe modified nucleotides. To optimize the molar ratio of N/P (the number of free amino groups in DPP to the number of nucleotides in ASOs) for the preparation, we evaluated DPP/ASOs nanoparticles (AD) prepared with the N/P molar ratio ranging from 1 to 16 by agarose gel electrophoresis. The results indicated that the ASOs were completely encapsulated by all DPPs except DPP10 and DPP12, when the N/P molar ratio was higher than 4 (Fig. 1b and Additional file 1: Fig. S4). As the electrostatic interaction is the main force between DPPs and ASOs, the fewer positive charges in DPP would lead to the weaker interactions, and ultimately the lower encapsulation efficacy. The N/P molar ratio = 8 was chosen for the preparation of AD and DP-AD in the following study to avoid high cytotoxicity and high clearance rate of NPs caused by high N/P molar ratio as reported previously [19, 30]. The encapsulation rates of ASOs were above 85% when the N/P molar ratio was 8 (Additional file 1: Fig. S5a, b). Then, we optimized the molar ratio of DSPE-mPEG2000. We had previously confirmed that AD7 could effectively deliver ASOs into bacteria when incubated in dd H_2_O (data not shown), while the delivery efficiency decreased significantly when treated with M–H broth (Additional file 1: Fig. S6a), which was caused by the aggregation of AD7 in the complicated medium (Fig. 1d). The flow cytometry results indicated that DP-AD7 got the highest delivery efficiency when the molar ratio of DSPE-mPEG2000 to ASO was 0.5: 1 (Additional file 1: Fig. S6a). Furthermore, the growth curve results showed that DP-AD7 with the same DSPE-mPEG2000 ratio (0.5: 1) had the highest inhibitory efficacy compared to the counterpart mismatched group (Additional file 1: Figure S6b–e). DSPE-mPEG2000 was a widely used phospholipids-polymer conjugate in drug delivery applications. It was a biocompatible, biodegradable and amphiphilic material which could be functionalized with various biomolecules for specific functions [31, 32]. In our DP-AD delivery system, lower ratio of DSPE-mPEG2000 would impair the stabilized efficacy of DSPE-mPEG2000, while higher ration would impair the cell membrane penetration efficacy of DP-AD. Then, we measured the binding efficiency of DSPE-mPEG2000 in DP-AD2, DP-AD3, DP-AD7 and DP-AD8, which were about 50% (Additional file 1: Fig. S5c, d).

Then, we examined the formation of the intermediate product AD by transmission electron microscopy (TEM) and dynamic light scattering (DLS). The results demonstrated that DPPs, except for DPP4, DPP5, DPP10 and DPP12, could form spherical AD, the diameters were ranging from 50 to 80 nm examined by TEM, or about 120 nm examined by DLS (Fig. 1c, d, Additional file 1: Fig. S7). AD4 and AD5 were undetectable by neither DLS (data unshown) nor TEM, indicating the unsuccessful formation of NPs by these two DPPs. The excessive hydroxyl groups in DPP4 and DPP5 made them have stronger hydrophilic interactions with the solvent than with the ASOs during the preparation of AD. By contrast, the DLS results of AD10 and AD12 showed notably larger sizes (> 200 nm) compared with the other ADs, probably because there were only 6 positive charges in DPP10 or DPP12, leading to the looser interaction between these two DPPs and ASOs than those of other DPPs and evident aggregation between NPs (Additional file 1: Fig. S7). Notably, the size of successfully prepared ADs increased sharply after being diluted with M–H broth (Fig. 1d), suggesting that these intermediate products were aggregated in complicated solutions. After being decorated by DSPE-mPEG2000 (the molar ratio of DSPE-mPEG2000 to ASOs was 0.5), the DP-AD products were virtually spherical (Fig. 1e, Additional file 1: Fig. S7) and exhibited high stability, because their size did not change greatly after dilution in 50% M-H broth (Fig. 1f). The zeta potential of DP-AD was about + 33 mV (Fig. 1f), which is desirable for nano-delivery systems [33]. DLS results showed that the size of DP-AD were 130–150 nm, showing a narrow distribution with the polydispersity index (PDI) less than 0.4, which were larger than the values measured by TEM (60–80 nm) (Fig. 1e and Additional file 1: Table S4), consistent with the previous evidences attesting that the hydrodynamic diameters measured by DLS were larger than the solid diameters measured by TEM [34, 35]. Notably, DLS and TEM results of the linear DP-AD (L-DP-AD) were consistent with those of DPP7 (Fig. 1d, f, Additional file 1: Fig. S7), indicating the successful preparation of L-DP-AD. Furthermore, we studied the serum stability of DP-AD. After being incubated with equal volume of 10% FBS, the encapsulation of ASOs did not decrease significantly, indicating that there was no significant collapse of the nanoparticles (Additional file 1: Fig. S8a, b). Then, we furtherly confirmed the size of the nanoparticle didn’t increase significantly after incubation with equal volume of 10% FBS (Additional file 1: Fig. S8c, d). These results showed that DP-AD could keep stable structure in FBS medium without observable collapse or heterogeneous aggregation. Given that DPP4, DPP5, DPP10 and DPP12 could not form stable nanoparticles with ASOs, they were excluded in the following experiments.

### Screening of DP-AD

**Fig. 2:**
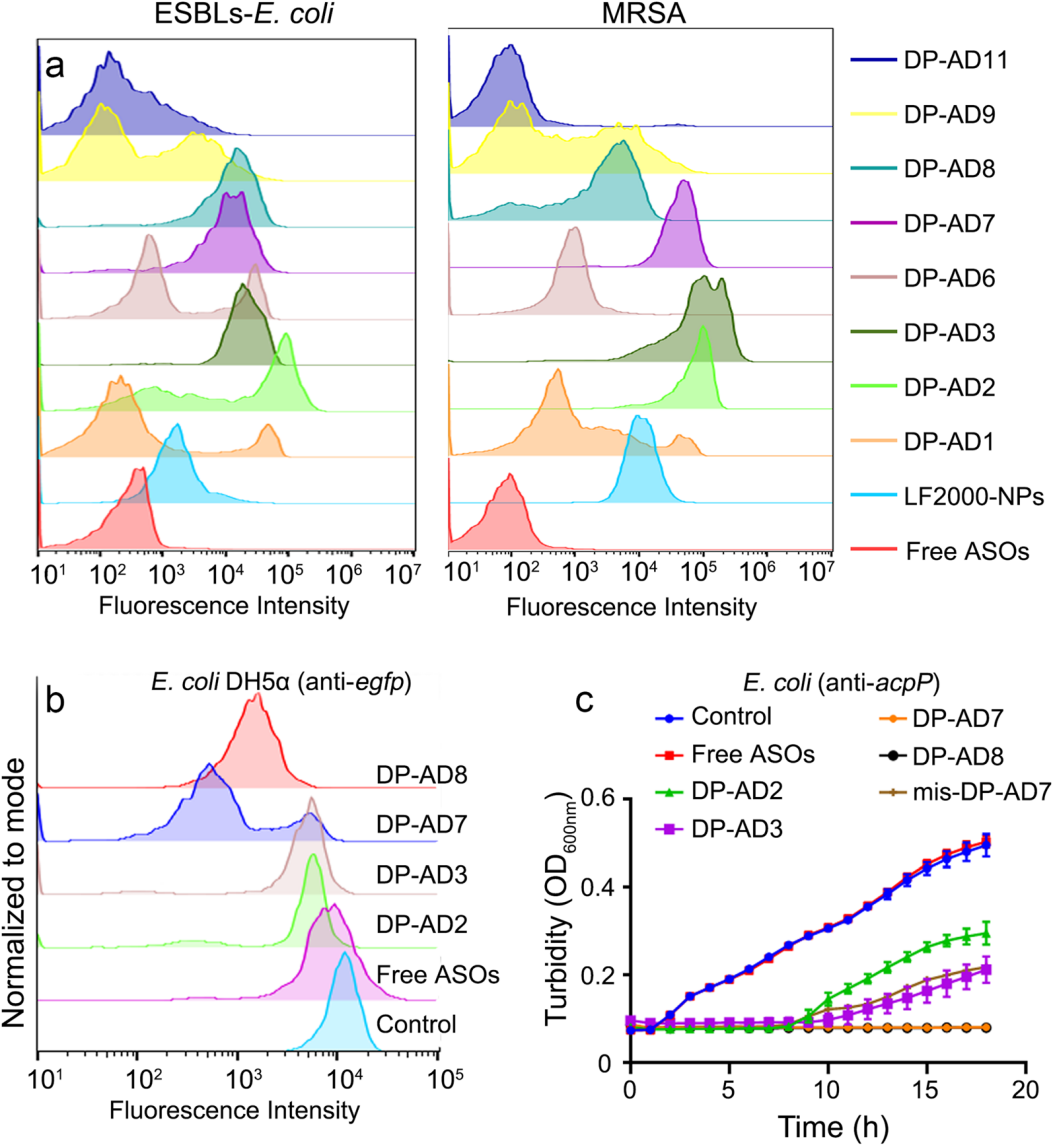
Screening the DP-AD by delivery efficiency and antisense efficacy in vitro.** a** FAM-positive ratio of ESBLs-*E. coli* (left) and MRSA (right) were tested by flow cytometry after incubation with FAM-labeled DP-AD for 1 h in dark at 37 °C. Free FAM-labeled ASOs (red) and LF2000-NPs (light blue) were used as negative and positive controls, respectively. **b** GFP fluorescence intensity of *E. coli* (DH5α) expressing the GFP measured by flow-cytometry after incubation with 1 μM DP-AD_anti-*egfp*_ for 3 h. **c** Growth curves of ESBLs-*E. coli* treated with different amphipathic DP-AD_anti-*acpP*_ (1 μM), OD_600nm_, the optical density at 600 nm

To explore the critical factors that determine the penetration efficacy of DPP and screen the DP-AD with the highest delivery efficacy in bacteria, the Gram-negative *E. coli* and ESBLs-*E. coli* and Gram-positive *S. aureus* and MRSA were incubated with FAM-labeled DP-AD at 37 °C for 1 h away from light. Then, the FAM-positive bacterial ratio was measured by flow cytometry. The results showed that free ASOs without any carrier could hardly be internalized by all the tested strains, while the amphipathic DP-AD2, DP-AD3, DP-AD7 and DP-AD8 showed an uptake efficiency of about 90% by the tested strains, significantly higher than that of commercially available transfection reagent LF2000-NPs which showed positive ratios about 50%–75%. On the other hand, the bacteria treated by hydrophilic DP-AD1 or DP-AD6 and amphipathic DP-AD9 or DP-AD11 with four His residues exhibited the lowest uptake efficiency of 10% to 45% (Fig. 2a, b, Additional file 1: Fig. S9 and Table S5), suggesting that the hydrophilicity of DPP and excessive His residues in DPP may hamper the uptake process of NPs. Both His and Arg residue contained a positive charge, but the mechanism for the impeding effect of excessive amount of His residues to the delivery efficiency of DP-AD needed further study. The failure of DPP10, DPP12 and L-DPP to deliver ASOs into *E. coli* and ESBLs*-E. coli* (Additional file 1: Fig. S9) further confirmed that, the amphipathic and dendritic structure of DPP and the positive charge numbers played key roles in the delivery process. Consistent with the above results, DPP4 and DPP5 lacked the capacity of ASOs delivery in the tested bacterial strains (Additional file 1: Fig. S10). Through this screening experiment, the amphipathic DP-AD2, DP-AD3, DP-AD7 and DP-AD8 with positive charges ≥ 8 possessed a high capability to deliver ASOs into bacterial strains and were selected for further experiment.

Although DP-AD2, DP-AD3, DP-AD7 and DP-AD8 had similar delivery capacity in the tested strains, whether these DP-AD could release the cargos and exert antisense inhibitory effects in bacteria remains unclear. Thus, we prepared DP-AD targeting *egfp* or *acpP* (DP-AD_anti*-egfp*_ or DP-AD_anti*-acpP*_) and tested their antisense inhibitory efficacy on *E. coli* expressing enhanced green fluorescent protein (EGFP) or ESBLs-*E. coli*, respectively. Flow cytometry showed that 1 μM DP-AD7_anti*-egfp*_ and DP-AD8_anti*-egfp*_ significantly reduced the GFP fluorescent intensity of bacteria compared with the control and mismatched groups. However, DP-AD2_anti*-egfp*_ and DP-AD3_anti*-egfp*_ with the same concentration showed no effects on the GFP fluorescent intensity (Fig. 2b). Consistently, the growth curve results showed that 1 μM DP-AD7_anti-*acpP*_ and DP-AD8_anti-*acpP*_ exhibited stronger inhibitory effects on the growth of ESBLs-*E. coli* than DP-AD2_anti-*acpP*_*,* DP-AD3_anti-*acpP*_*,* or the mismatched group (DP-AD7_mismatch_) (Fig. 2c). These data furtherly confirmed that the presence of two His residues in DPP was beneficial to the transfection efficiency of ASOs in bacteria by DP-AD. As DP-AD2, DP-AD3 had the similar delivery efficiency with DP-AD7 and DP-AD8, it is reasonable to infer that His residues may promote the release of ASOs from DP-AD7 and DP-AD8 after entering bacterial cells. These results were in accordance with a previous study demonstrating that the “proton-sponge effect” of His residues in CPPs promotes the escape of NPs from endosomes, thus enhancing the transfection efficiency in host cells [24, 36]. However, as far as we knew, it was unclear that whether bacteria had endosomes or the similar structures in mammalian cells, which is an open question for further research. We also observed evident antibacterial effects of 1 μM DP-AD7_mismatch_ compared with the control or free ASOs groups, indicating DPP7 also exhibited certain antibacterial activity. To further validate the antibacterial activity of DPPs, minimum inhibitory concentration (MIC) assays revealed that all the amphipathic DPPs (DPP2, DPP3, DPP7 and DPP8) showed antibacterial activity against *E. coli*, ESBLs-*E. coli, S. aureus* and MRSA. And the MICs of DPP7 and DPP8 for the tested bacteria were 16–32 μg mL^−1^ (Additional file 1: Table S6). The antibacterial activity of these DPPs would be caused by the positive charge of DPPs, which can be further modified to enhance their antibacterial activity and develop bifunctional antibacterial agents in the future. We also measured the cytotoxicity of DP-AD2, DP-AD3 and DP-AD7, the results showed that free DPPs and DP-AD showed no observable cytotoxicity in normal human small intestine epithelial cells (HIEC) (Additional file 1: Fig. S11a). In addition, we also investigated the hemolytic activity of these nanoparticles. The results demonstrated that when the concentration was no more than 1 μM, the maximum concentration in our experiments, the nanoparticles showed no observable hemolytic activity in red blood cells (Additional file 1: Fig. S11b). Nevertheless, it was noted that when the concentration was up to 2 μM, DP-AD2 and DP-AD3 showed much higher hemolytic toxicity than DP-AD7 and DP-AD8.

### The delivery efficacy of DP-AD and its bacteria spectrum for delivery

Given the results that DP-AD7 and DP-AD8 had similar delivery profiles and antisense efficacy, DP-AD7 was selected as the representation to study their antibacterial efficacy in vitro and in vivo.

**Fig. 3:**
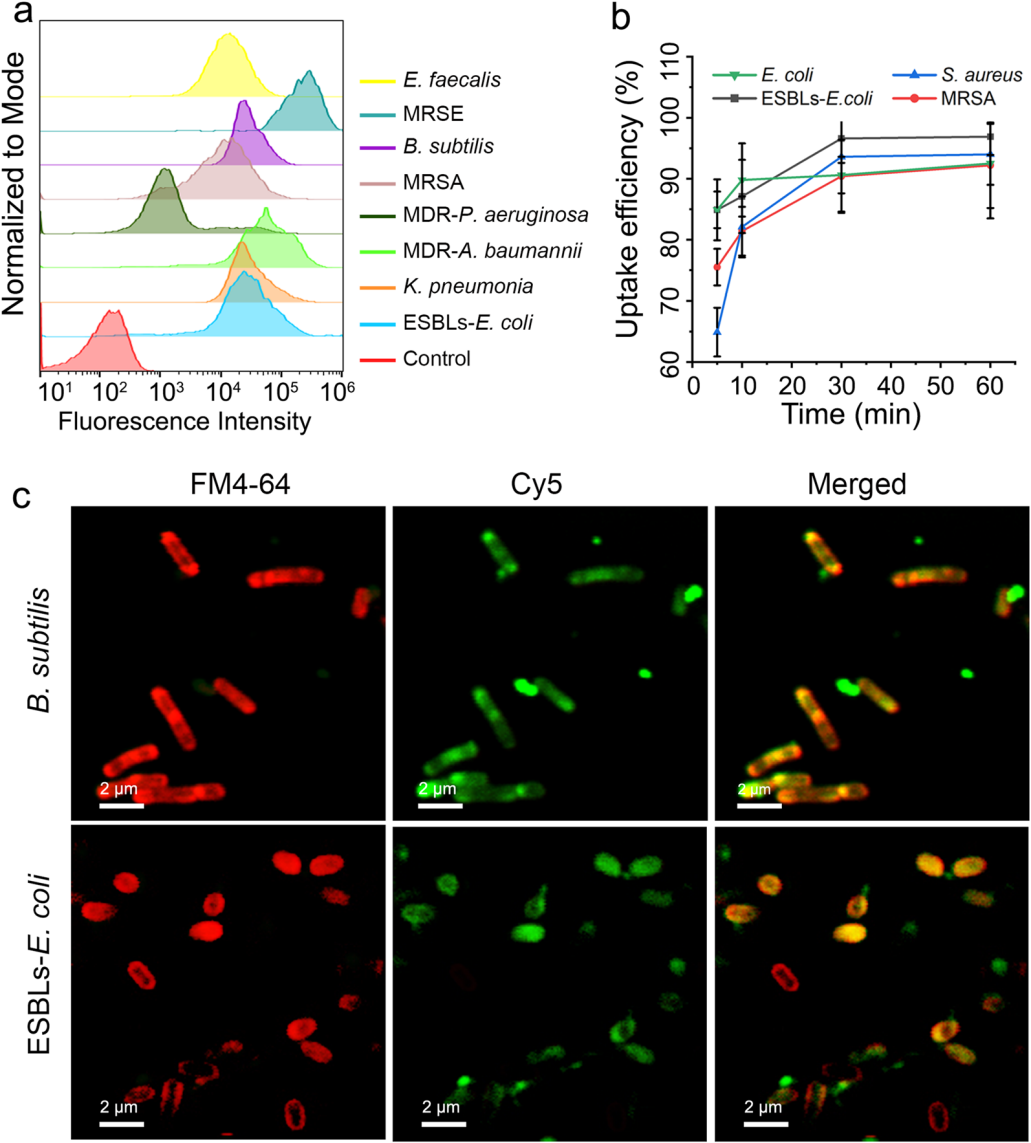
Uptake profiles of DP-AD7. **a** Fluorescence positive ratios of ESBLs-*E. coli*, *K. pneumoniae*, MDR-*A. baumannii*, MDR-*P. aeruginosa*, MRSA, *B. subtilis*, MRSE and *E. faecalis* measured by flow-cytometry after incubation with 1 μM FAM labeled DP-AD7 for 1 h in dark. **b** The FAM positive rate of the tested bacterial strains measured by flow cytometry after incubation with FAM-labeled DP-AD7 in the dark at 37 °C for 5, 10, 30 and 60 min. **c** CLSM images of ESBLs-*E. coli* (upper panel) and *B. subtilis* (lower panel) after incubation with cy5-labeled DP-AD7 for 1 h in dark at 37 °C. FM4-64 was used to stain the bacterial cell membrane. Bar = 2 μm

Firstly, we explored the delivery potential of DP-AD to carry ASOs into different bacterial strains, FAM-labeled DP-AD7 was incubated with Gram-negative ESBLs-*E. coli* (ATCC35218), *K. pneumonia* (ATCC75293), MDR-*A. baumannii* (XJ17014279) and MDR-*P. aeruginosa* and Gram-positive MRSA, *B. subtilis* (ATCC23857), MRSE and *E. faecalis* (ATCC29212), respectively. Then the fluorescent positive ratios were measured by flow cytometry. The results showed that all the tested strains had a positive ratio higher than 85% with different fluorescent intensities (Fig. 3a), indicating DP-AD7 could successfully deliver ASOs into all the tested bacterial strains, including *P. aeruginosa*, which was intrinsic resistant to many kinds of small molecular antibiotics because of the poor permeability of the outer membrane [37].

Then we further explored the dynamic features of the uptake process of DP-AD7 in different bacterial strains. FAM-labeled DP-AD7 were incubated with ESBLs-*E. coli*, *E. coli, S. aureus* and MRSA for 5, 10, 30 and 60 min, respectively. Then FAM-positive bacterial ratio was measured. The results showed that the FAM-positive bacterial ratio reached the peak value at 10 min in *E. coli* and ESBLs-*E. coli*, while at 30 min in *S. aureus* and MRSA (Fig. 3b). These results indicated that DP-AD7 could be internalized rapidly in both Gram-negative and Gram-positive bacteria. As the Gram-positive bacteria had thicker cell wall than Gram-negative bacteria, we hypothesized that the cell wall had a higher blocking influence on the delivery efficiency than the cellular membrane. To further ensure the delivery efficiency, bacteria and NPs were incubated at different temperatures. As a result, no significant difference was observed between the uptake efficiency of DP-AD7 when incubated at 37 °C or 4 °C by all the four tested strains (Additional file 1: Fig. S12), implying that the uptake process by bacteria is not dependent on temperature related processes, such as energy state [38, 39]. However, the concrete uptake mechanism of DP-AD7 and the influence of the cell wall on the delivery process required further study.

To further confirm the potential of DP-AD7 to carry ASOs into bacteria, confocal laser scanning microscopy (CLSM) was adopted to observe the internalization of DP-AD7 by ESBLs-*E. coli* and *B. subtilis*. After incubation with cy5-labeled DP-AD7 in dark for 1 h, strong green fluorescence was observed in the cytoplasm of both ESBLs-*E. coli* and *B. subtilis* (Fig. 3c), in contrast to the red fluorescence staining in the bacterial cell membrane labeled with FM4-64, indicating that DP-AD7 could be successfully internalized by the tested strains. From these data, DP-AD7 could efficiently deliver ASOs into a broad spectrum of bacteria, including the well-known MDR-*A. baumannii* and MDR-*P. aeruginosa,* which are intrinsic resistant to most antibiotics because of their low penetration of the outer membrane.

### Antisense antibacterial activity of DP-AD7 in vitro and in vivo

**Fig. 4:**
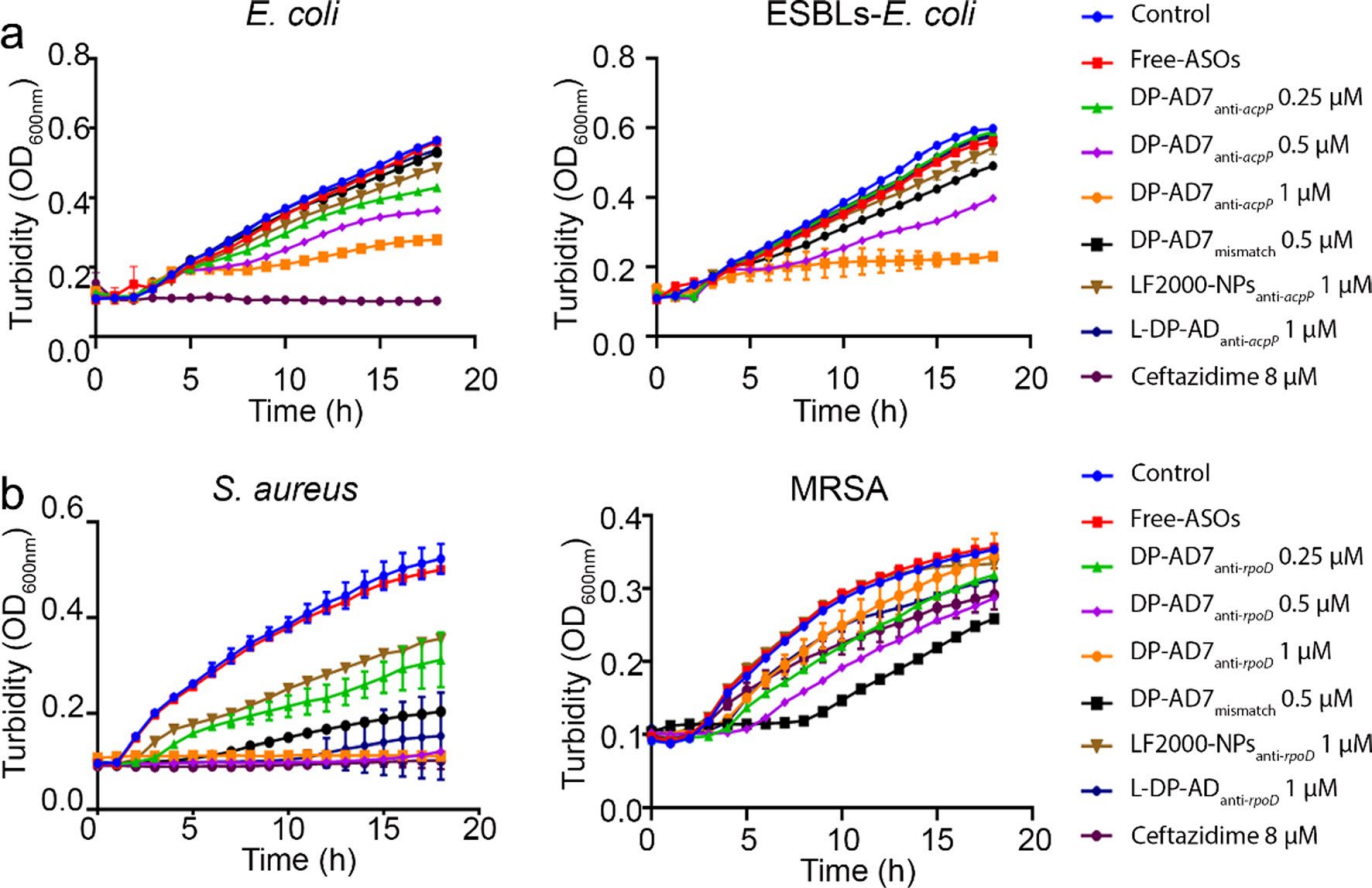
Antibacterial activity of DP-AD7 in vitro. **a** Growth inhibition of DP-AD7_anti-*acpP*_ on *E. coli* (left) and ESBLs-*E. coli* (right) or **b** DP-AD7_anti-*rpoD*_ on *S. aureus* (left) and MRSA (right) were measured. Ceftazidime and LF2000-NPs_anti-*acpP*_ were used as positive control, and free ASOs and L-DP-AD were used as negative control. OD_600nm_, the optical density at 600 nm

Then, we studied the antibacterial activity of DP-AD7 in vitro and in vivo. We first studied the antisense inhibitory effect of DP-AD7_anti-*acpP*_ and DP-AD7_anti-*rpoD*_ on *E. coli* and ESBLs-*E. coli*, *S. aureus* and MRSA, respectively. The results showed that DP-AD7_anti-*acpP*_ and DP-AD7_anti-*rpoD*_ significantly inhibited the growth of the tested bacterial strains in a concentration dependent manner, showing a higher inhibition efficacy than those treated with DP-AD7_mismatch_ or LF2000-NPs (Fig. 4). Consistent with the above results that DPP7 had intrinsic antibacterial effect, the 1 or 0.5 μM DP-AD7_mismatch_ also showed a significant inhibitory effect compared with the control group (Additional file 1: Fig. S13). When treated with 0.5 or 1 μM DP-AD7, the knockdown efficiency of *acpP* in *E. coli* and ESBLs-*E. coli* and *rpoD* in *S. aureus* and MRSA were much higher than that of the mismatched counterpart groups as revealed by RT-PCR assay (Additional file 1: Fig. S14). Importantly, DP-AD7 showed a stronger inhibition effect of gene expression compared with commercial LF2000-NPs. The linear counterpart, L-DP-AD_anti-*acpP*_ or L-DP-AD_anti-*rpoD*_, showed neither growth inhibition nor gene suppression effect on the tested bacterial strains. These results demonstrated that DP-AD7 could significantly inhibit the bacterial growth and expression of target gene in vitro.

**Fig. 5:**
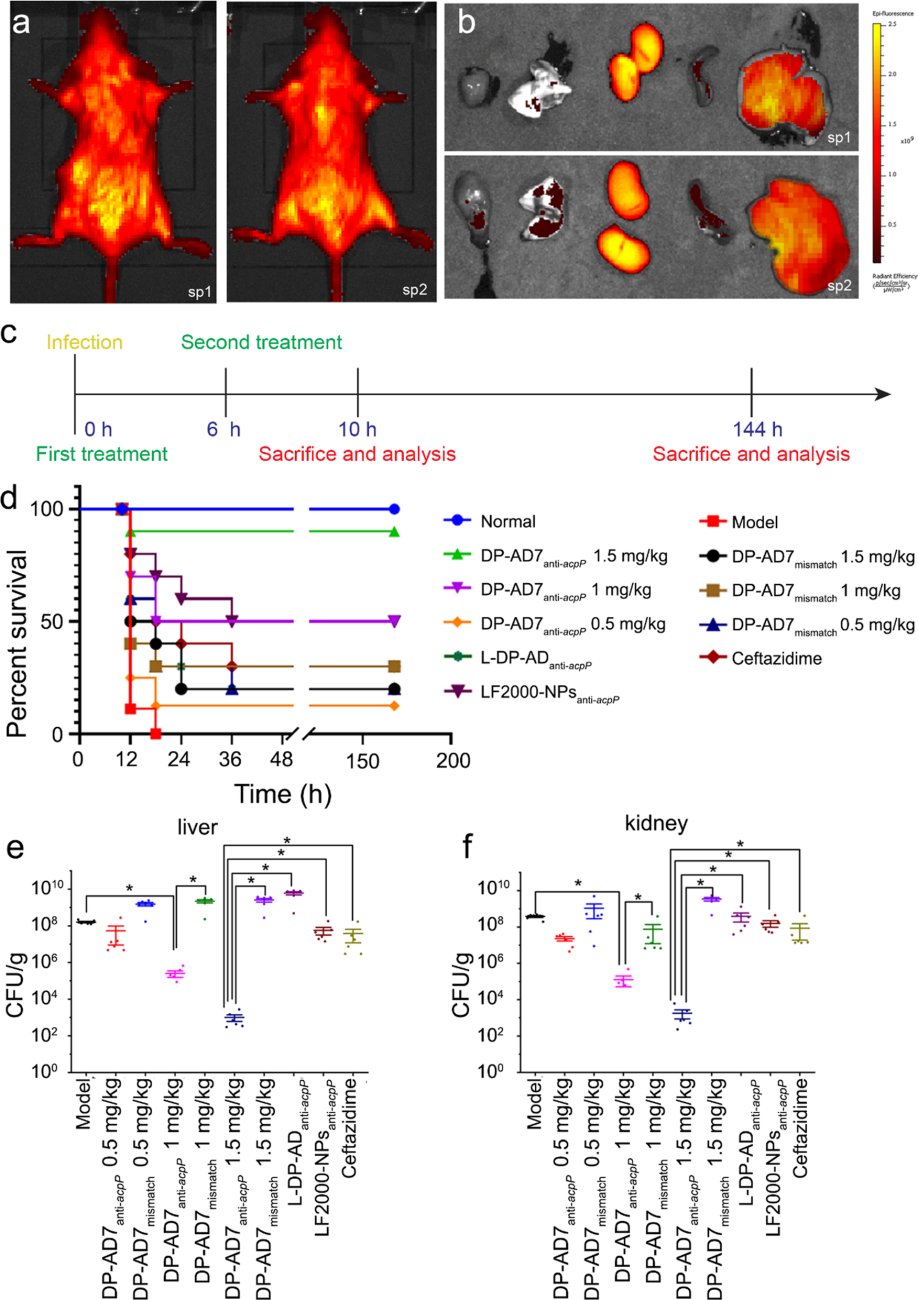
Antibacterial activity of DP-AD7_anti-*acpP*_ against ESBLs-*E. coli* in the sepsis model. **a**, **b** Biodistribtution of DP-AD7_anti-*acpP*_ in mice determined by in vivo imaging system. Mice were intraperitoneally administrated with 400 μl cy5-labeled DP-AD7. Fluorescent signals were detected in the live mice (**a**) or the collected organs of the mice (**b**) 2 h after injection. sp1 and sp2 were two independent samples. **c** The diagram of treatment and analysis procedure of the in vivo experiment. **d** Survival rate of BalB/c mice treated with DP-AD7_anti-*acpP*_ (1.5, 1 and 0.5 mg/kg), DP-AD7_mismatch_ (1.5, 1 or 0.5 mg/kg), or L-DP-AD_anti-*acpP*_ (1.5 mg/kg) (n = 10). LF2000-NPs_anti-*acpP*_ (1.5 mg/kg) and ceftazidime (4 mg/kg) were used as positive controls. **e**, **f** Colonization of ESBLs*-E.coli* inocula in liver (**e**) and kidney (**f**). Data represent the mean ± SE (n = 6). *p < *0.05*

To explore the in vivo effect of DP-AD7, we firstly determined the distribution of DP-AD7 in mice by an in vivo imaging system. As a result, DP-AD7 was mainly distributed in the liver and kidney of mice 2 h after intraperitoneal injection of cy5-labeled DP-AD7 (1.5 mg kg^−1^, amounting to 225 nmol kg^−1^ ASOs) (Fig. 5a, b). Then, we studied the antibacterial efficacy of DP-AD7_anti-*acpP*_ in vivo. The mouse sepsis model was established by intraperitoneal administration of ESBLs-*E. coli* (4 × 10^5^ CFU in 0.4 mL M–H broth) into each mouse (Male BALB/c, 8–10 weeks of age and weighing 18–22 g). These infected mice received different treatments (Fig. 5c), including control agents, different doses of DP-AD7_anti-*acpP*_, DP-AD7_mismatch_, L-DP-AD_anti-*acpP*_, LF2000-NPs_anti-*acpP*_ and ceftazidime. All the mice infected with ESBLs-*E. coli* in the model group died within 24 h. The intraperitoneal administration of DP-AD7_anti-*acpP*_ with doses ranging from 0.5 to 1.5 mg kg^−1^ (amounting to 75 to 225 nmol kg^−1^ ASOs, respectively) significantly improved the mice survival rate in a dose-dependent manner. The survival rates of mice treated with 1 or 1.5 mg kg^−1^ DP-AD7_anti-*acpP*_ were improved to 50% and 90%, respectively. These values were significantly higher compared with that of DP-AD7_mismatch_ treated groups, which had 30% and 20% survival rates in 1 or 1.5 mg kg^−1^ groups, respectively. The higher concentration of DP-AD7_mismatch_ showed lower survival rate may be caused by the hemolytic toxicity of DP-AD7 (Additional file 1: Fig. S11b). Notably, the mice treated with 1.5 mg kg^−1^ LF2000-NPs_anti-*acpP*_ or ceftazidime had a survival rate of 50% or 30%, respectively, significantly lower than that of the DP-AD7_anti-*acpP*_ treated group. Consistent with the results in vitro, DP-AD7_mismatch_ also showed protective effects on infected mice. The survival rate of the mice treated with L-DP-AD7_anti-*acpP*_ was only about 20% (Fig. 5d). The increased survival rate was associated with a reduction of the bacterial load in the liver and kidney, where the NPs mainly distributed (Fig. 5a, b), of mice inoculated with ESBLs-*E. coli*. Ten hours after infection, the average bacterial load in the kidney was 3.77 ± 1.07 × 10^8^ CFU g^−1^ in the model group, which was reduced to 1.24 ± 1.80 × 10^5^ or 1.80 ± 2.28 × 10^3^ CFU g^−1^ in the group treated with 1 or 1.5 mg kg^−1^ DP-AD7_anti-*acpP*_, respectively. The bacterial load values in the DP-AD7_anti-*acpP*_ treated groups were significantly lower than that of DP-AD7_mismatch_ treated groups, which showed 7.39 ± 1.51 × 10^7^ and 3.40 ± 1.77 × 10^9^ CFU g^−1^, in mice groups treated with 0.5 and 1.5 mg kg^−1^ DP-AD7_mismatch_, respectively. Meanwhile, the CFU number in the groups treated with 1.5 mg kg^−1^ LF2000-NPs_anti-*acpP*_, L-DP-AD_anti-*acpP*_ or ceftazidime were 1.58 ± 1.49 × 10^8^, 3.85 ± 4.80 × 10^8^ or 8.41 ± 1.60 × 10^7^ CFU g^−1^, which were significantly higher than those in the DP-AD7_anti-*acpP*_ treated group (Fig. 5e). Similar CFU results were observed in the liver of the infected mice (Fig. 5f). Pathological analysis of the animal tissues indicated that, DP-AD7_anti-*acpP*_ treatment significantly attenuated the lesion and congestion of the liver and kidney compared with those treated with DP-AD7_mismatch_ or ceftazidime (Additional file 1: Fig. S15). Collectively, these results confirmed the strong antibacterial activity of DP-AD7_anti-*acpP*_ in mice infection model.

## Conclusions

We designed a series of DPPs with different hydrophobicity, positive charge distribution pattern and amino acid types, and prepared a novel type of DP-AD non-covalently. We found that hydroxyl groups in DPPs and a positive charge number less than 8 would interfere the formation of NPs. The centralized distribution of positive charges could facilitate the delivery of DP-AD, but an averaged distribution of positive charge would hamper this process. The amphipathic DPP2, DPP3, DPP7 and DPP8 showed a notably higher efficiency to deliver ASOs into Gram-negative and Gram-positive bacteria, whereas only amphipathic DPP7 and DPP8 with two His residues in sequence exhibited the best antisense inhibitory efficiency in vitro. Importantly, DP-AD7_anti-*acpP*_ showed excellent antibacterial activity in mice infected by ESBLs-*E.coli*. Based on these results, the hydrophilic terminals in the DPPs with 8–12 positive charges are beneficial for complexion with ASOs. The hydrophobic terminals facilitate DPPs entering bacteria by strengthening their interactions with lipid-rich plasma membrane, whereas two His residues in DPPs promote the release of the loaded drug in the intracytoplasmic of bacteria. In conclusion, we determined for the first time the key factors influencing the formation of DP-AD and the transfection efficiency in bacteria, and provide a novel approach for further research and application of antisense antibacterial strategy.

## Methods

### Materials

DPPs (Scheme 1, Additional file 1: Table S1, S2 and Fig. S1–S3) were synthesized by ChinaPeptides Co., Ltd. (Suzhou, China). 2′-O-methyl (2′-OMe) modified antisense oligonucleotides (ASOs), 5′-fluorescein amidites (FAM)-labeled ASOs and primers were synthesized by Sangon Biotech (Shanghai, China) (Additional file 1: Fig. S3). 5′-Cyanine 5 (Cy5)-labeled ASOs were synthesized by Tsingke Biological Technology Co., Ltd (Xi’an, China). 1,2-distearoyl-sn-glycero-3-phosphoethanolamine-N-methoxy(polyethylene glycol)-2000 (DSPE-mPEG2000) was purchased from Xi'an ruixi Biological Technology Co., Ltd. Trizol (Additional file 1: Fig. S3), FM4-64 dye, Lipofectamine 2000™ and Opti-MEM medium were purchased from Life Technologies (Invitrogen, CA, USA). Lysogeny broth and Mueller–Hinton (M–H) broth were purchased from Beijing Land Bridge Technology Co., Ltd (Beijing, China). Reversed enzyme and SYBER Green enzyme were purchased from Takara Bio Inc. (Kyoto, Japan).

### Bacterial strains and cell line

*Escherichia coli* (*E. coli*, ATCC25922), extended spectrum β-lactamase-producing *Escherichia coli* (ESBLs-*E. coli*, ATCC35218), *Klebsiella pneumonia* (*K. pneumonia,* ATCC75293), multidrug resistant *Pseudomonas aeruginosa* (MDR-*P. aeruginosa*), *Staphylococcus aureus* (*S. aureus*, ATCC29213), *Escherichia faecalis* (*E. faecalis*, ATCC29212) and *Bacillus subtilis* (*B. subtilis*, ATCC23857) were stored in our laboratory, methicillin-resistant *Staphylococcus epidermidis* (MRSE), methicillin-resistant *Staphylococcus aureus* (MRSA), Multidrug-resistant *Acinetobacter baumannii* (MDR-*A. baumannii* XJ17014279) was isolated from Xijing Hospital, Fourth Military Medical University. *E. coli* (DH5α) were obtained from Beijing Beina Chuanglian Biotechnology Institute.

### Nanoparticles (NPs) preparation

LF2000-NPs were prepared in strict accordance with the protocol described in our previous study [40]. DPP storing solutions (Additional file 1: Table S3) were diluted 50 times to obtain the working solutions. DSPE-mPEG2000 decorated DPP/ASOs nanoparticles (DP-AD) were prepared in two steps. Firstly, the DPP working solution (3 μl) and ASOs (15 μl, 20 μM) dissolved in RNAse/DNAse free sterile water (0.02 mM) were diluted to 275 μl by RNAse/DNAse free sterile water and mixed at 2600 rpm for 1 min, followed by incubation at 37 °C for 30 min to form DPP/ASOs nanoparticles (ADs). Secondly, 7.5 μl DSPE-mPEG2000 solution (20 μM, 0.5-fold of ASOs) was added into the ADs solutions, and the mixture was mixed at 2600 rpm for 1 min and incubated at 37 °C for another 30 min to obtain the DP-AD solution (1 μM), whose concentration was calculated based on the concentration of ASOs. DP-AD with different DSPE-mPEG2000 ratios were prepared with the same procedure with different DSPE-mPEG2000 volumes. The linear DP-AD (L-DP-AD) were prepared with the same procedure used for DP-AD.

### Agarose gel electrophoresis

The ASOs solution was incubated with DPPs at 37 °C for 30 min at geometric N/P molar ratios ranging from 0 to 16 to form ADs. The prepared ADs (10 μl, 40 μM) were analyzed by electrophoresis on agarose gel (1% wt/vol) and stained with ethidium bromide to obtain the images.

### Transmission electronic microscope (TEM)

TEM was adopted to observe the morphology of the NPs. Briefly, a drop of NPs solution (30 μM) was added to slide-grids, followed by natural settling for 5 min in ambient conditions before the liquid was sucked away quickly. Then, the grids were dried in ambient conditions. Images were captured using JEM-1230 Electron Microscope (JEOL, Japan) at 80 kV.

### Size and zeta potential measurement

Size and zeta potential of DP-AD were measured by zeta sizer (Marlvern Panalytical, UK). The NPs solution (1 ml) was added into a disposable cuvette with an optical path of 1 cm. The measurement conditions for size were as following: the dispersant was water; Mark-Houwink parameters were 0.428 (A parameter) and 7.67 × 10^–5^ (K parameter); measurement temperature was 25 °C; the measurement angle was 173°. The NPs solution (approximately 1 ml) was added into a disposable folded capillary cell, followed by the measurement of the zeta potential at 25 °C. The size of DP-AD in M–H broth were measured after 500 μl NPs solution were diluted with an equal volume of M–H broth. The analysis was performed in triplicate by Zetasizer software (version 7.13, Malvern).

### Bacterial culture

Bacteria stored in 15% glycerin were streaked onto M–H agarose plate and cultured at 37 °C for 18 h. Next, a bacterial colony was transferred from the M–H agarose plate into Lysogeny broth (LB, 3 ml) in quartz tubes and cultured at 37 °C until reaching the logarithmic growth stage.

### Flow cytometry analysis

To measure the delivery efficiency, FAM-labeled DP-AD were added to bacterial cultures (approximately 5 × 10^6^ CFU in 300 μl M–H broth) and incubated at 37 °C for 1 h away from light. The bacterial solutions were centrifuged at 2500 g at 4 °C for 5 min and washed twice with phosphate buffered saline (PBS) before analyzing them using the BL1 (green) channel in flow cytometry (Novocyte, Acer, USA). Data were analyzed using Flowjo software (version 10.0.7, Tree Star, Ashland, OR, USA). Free FAM-labeled ASOs and LF2000-NPs were used as the negative and positive control, respectively.

To measure the delivery rate of DP-AD7 in different types of bacteria, the bacteria (approximately 5 × 10^6^ CFU in 300 μl M–H broth) were incubated with FAM-labeled DP-AD7 for 5, 10, 30 or 60 min at 37 °C or at 4 °C for 60 min in dark, then the bacterial solutions were processed, measured and analyzed as described above.

*E. coli* (DH5α), an engineering bacterial strain expressing EGFP, was adopted to measure the antisense efficacy of DP-AD in vitro. 3 × 10^6^ CFU bacteria in 300 μl M–H broth were treated with 300 μl amphipathic DP-AD2, 3, 7 or 8 solutions (1 μM) at 37 °C for 1 h. Then, fluorescence intensity was measured by flow cytometry and analyzed as described above.

The FAM positive rates of ESBLs-*E. coli* (ATCC35218), *K. pneumoniae* (ATCC75293), MDR-*P. aeruginosa*, *E. faecalis* (ATCC29212), *B. subtilis* (ATCC23857), MRSE, MRSA and MDR-*A. baumannii* (XJ17014279) were measured as following: 3 × 10^6^ CFU bacteria in 300 μl M–H broth were incubated with 300 μl DP-AD7 at 37 °C for 1 h. Then, fluorescence intensity was measured by flow cytometry and analyzed as described above.

### Confocal laser scanning microscope (CLSM)

Firstly, cy5-labeled DP-AD7 solution (300 μl, 2 μM) was incubated with ESBLs-*E. coli* or *B. subtilis* (10^7^ CFU in 300 μl M–H broth) for 1 h at 37 °C away from light. Secondly, the bacterial solutions were centrifuged at 2500 g for 5 min to discard the supernatant, and washed twice with 500 μl PBS, followed by resuspension of the bacteria with 20 μl PBS. Then, 1 μl FM4-64 dye was added into the bacterial solutions to stain the plasma membrane at ice for 1 min, then, the solutions were centrifuged at 2500 g for 5 min, discarded the supernatant, and resuspended the bacteria with 20 μl PBS. Then, several drops of these solutions were added onto a 0.5-cm cover glass and dried at 37 °C, followed by the addition of a drop of glycerin 50% to fix the bacteria. Lastly, CLSM was adopted to measure bacterial fluorescence. Images were captured and analyzed by Olympus Fluoview Viewer (version 3.0, Olympus Corp., Japan).

### Serum stability of DP-AD

500 μl FAM-labeled DP-AD (1 μM) was mixed with equal volume of 10% fetal bovine serum (FBS, DMEM as the medium) for 1 h at 37 °C, the DLS was used to measure the size of DP-AD. Then, the nanoparticles were centrifuged at 13,000 *g* for 30 min, then the fluorescence intensity of the supernatant from each DP-AD was measured. And the release of the ASOs from nanoparticles was measured. The DP-AD treated with equal volume of DMEM without FBS was used as the control. 1 ml FAM-labeled DP-AD (1 μM) was mixed with equal volume of 10% FBS for 6 h at 37 °C, then the size and PDI were measured as described above.

### Minimum inhibitory concentration (MIC)

DPP bacterial toxicity was evaluated by MIC assay of DPPs in *E. coli*, ESBLs-*E. coli, S. aureus* and MRSA. Briefly, DPP (50 μl, with a geometric concentration ranging from 64 μg ml^−1^ to 0.5 μg ml^−1^ dissolved in M–H broth) was added into 96-well plates, followed by the addition of an equal volume of the tested bacterial solutions (approximately 10^6^ CFU). Then, the mixture was incubated at 37 °C for 24 h and the optical density was measured using Bio-Rad 680 microplate reader (BioRad Corporate, Hercules, California, USA).

### Hemolytic assay

1 ml blood (Drawn from Zhou Chen) was centrifuged with 1000 rpm for 5 min to pellet the red blood cells, which was washed twice with 1 ml PBS. Then, the cells were resuspended with 1 ml PBS, followed by dilute by PBS to get 4% red blood cells solution. Then, 200 μl cell solution was mixed with the equal volume of DP-AD with the concentration ranging from 0.25 μM to 2 μM in 1.5 ml tube. After 1 h of incubation at 37 °C, the solutions were centrifuged at 1000 rpm for 5 to pellet the unlysed cells, 100 μl liquid from each tube was transferred to a clean 96-well plate. The optical density of 540 nm was measured. Finally, the percent hemolysis in each assay was calculated. PBS or 1% triton was used as negative and positive control, respectively.

### Mammalian cellular toxicity

The cytotoxicity of DPPs and DP-AD were investigated on HIEC cells. A total of 60,000 cells plated in 96-well plates the day before transfection were incubated with 1 μM of DP-AD and equivalent DPPs solutions for 24 h. Cytotoxicity was measured by colorimetric MTT assay (Sigma, Germany). Cell culture medium was removed and replaced with PBS containing 2.5 mg/ml of MTT for 4 h.

### mRNA quantification

Bacterial solutions (approximately 3 × 10^7^ CFU in 300 μl M–H broth) were incubated with DP-AD (300 μl, 3 μM) at 37 °C for 3 h. Bacterial total RNA was extracted using Trizol (Invitrogen, CA, USA). RNA was reversely transcribed to cDNA using HiScript™ Reverse Transcriptase (Vazyme Biotech co., Ltd) and mRNA was quantified using SYBR green detection kit following the manufacturer’s instructions with a Real-Time Q-PCR System (Mx3005p, Agilent Technologies StrataGene, La Jolla, CA, USA). The antisense sequences were 5′-cttcgatagtg-3′ for *acpP* [37, 41], 5′-acagctcctcgcccttcg-3′ for *egfp* in *E. coli* and ESBLs-*E. coli*, while 5′-tttctcgtca-3′ for *rpoD* in *S. aureus* and MRSA. The primers used were the following: *E. coli 16 s ribosomal RNA (rRNA)* forward 5′-cggacgggtgagtaatgt-3′ and reverse 5′-gtgcttcttctgcgggta-3′; *acpP* forward 5′-gagaattcatgagcactatcgaagaac-3′ and reverse 5′-agttaagcttgaccgcctggagatgttc-3′. *S. aurues 16 s rRNA* forward 5′-cgtggataacctacctataagact-3′ and reverse 5′-gattccctactgctgcctc-3′, *rpoD* forward 5′-cagatactgacgagaaa-3′ and 5′- gaataacataccacgac-3′. The PCR results in the tested strains were normalized using *16 s rRNA* as the housekeeping gene. Results were presented as the average fold change relative to the untreated control group by 2^−ΔΔCt^ method.

### Bacterial growth curve

*E. coli* solution in LB was centrifuged at 2500 g for 5 min at 4 °C and resuspended in M–H broth. Bacterial solutions (10^6^ CFU in 300 μl M–H broth) were mixed with 300 μl DP-AD_anti-*acpP*_ (*E. coli* and ESBLs-*E. coli*) or DP-AD_anti-*rpoD*_ (*S. aureus* or MRSA) solutions. The mixtures were divided into three wells on a culture plate and incubated into BioScreen C analyser at 37 °C for 18 h to measure the optical density per hour. Data were plotted and analyzed by Graphpad Prism (version 5.00, GraphPad Software, lnc., La Jolla, CA, USA).

### In vivo fluorescent image acquisition

400 μl DP-AD7 was administered into male Balb/c mice intraperitoneally, and the fluorescent images of the mice were acquired after 2 h. Then, the mice were sacrificed and the fluorescent images of the organs (heart, liver, spleen, lung and kidney) were acquired by an in vivo imaging system.

### Survival assay of DP-AD7 on infected animals

Male Balb/c mice (6–8 weeks old and weighing 18–22 g) were used in this experiment. The experimental and animal care procedures were approved by the Animal Care and Use Committee of Fourth Military Medical University. All methods were carried out strictly in accordance with the approved guidelines. 4 × 10^5^ CFU ESBLs-*E. coli* in 400 μl M–H broth were administrated intraperitoneally into the mice to construct a sepsis model. Then, the mice were randomly divided to ten groups, which were administered intraperitoneally with 400 μL solutions containing 0.5 (based on ASOs), 1 or 1.5 mg kg^−1^ DP-AD7_anti-*acpP*_ and the counterpart DP-AD7_mismatch_, 1.5 mg kg^−1^ L-DP-AD7_anti-*acpP*_, 1.5 mg kg^−1^ LF2000-NPs_anti-*acpP*_, 4 mg kg^−1^ ceftazidime or PBS at 0.5 and 6 h after infection. The surviving mice in each group were monitored every 6 h for the first day, afterwards, monitored every 12 h for 7 days day after infection, and the survival ratio was analyzed by Kaplan–Meier estimator.

Two mice in each group were sacrificed 10 h after infection to study the bacterial clearance. The liver and kidney were harvested aseptically, one kidney and one lobe of liver of each mouse were weighed, and homogenized in 500 μl sterile saline solution, then 50 μl homogenate sample of each mice was performed serial tenfold dilutions to a 10^–5^ dilution. Carefully spread 100 μL of each dilution onto a M–H agarose plate using a glass spreader. Incubate plates at 37 °C overnight and calculate the bacterial load. Colony counts were expressed as CFU g^−1^ of tissue.

The other kidney and liver lobe of each mouse were stored in 4% paraformaldehyde, paraffin embedding and hematoxylin and eosin staining were performed to observe the tissue lesions. The staining processes of tissues were carried out by the technicians in Department of Pathology, Fourth Military Medical University. The images were capture on Olympus BX15 with DP controller (version 3.1.1.267, Olympus Corp., Japan).

### Statistical analysis

Results are expressed as mean values (± SE). Statistical analyses were performed with SPSS (version 20.0.0, IBM Corp., Armonk, NY, USA). Differences between two groups were compared using *t* tests, and groups of two or more were compared to the control group using Dunnett *t* tests.

## Supplementary Information

**Additional file 1: Figure S1.** The HPLC and mass spectra of DPP1-DPP6. **Figure S2.** The HPLC and mass spectra of DPP7-DPP12. **Figure S3.** The identification of L-DPP, ASOs, and DSPE-mPEG2000. **Figure S4**. The screening of the N/P ratio of ADs. **Figure S5.** The encapsulation of ASOs and binding rate of DSPE-mPEG2000 in the nanoparticles. **Figure S6.** Screening the molar ratio of DSPE-mPEG2000 in DP-AD. **Figure S7**. The characteristics of the nanoparticles. **Figure S8. The serum stability of DP-AD. Figure S9.** Uptake efficiencies and growth curves of DP-AD in *E. coli* and *S. aureus*. **Figure S10.** Uptake profiles of DP-AD by *E. coli* and *S. aureus*. **Figure S11. The cytotoxicity and hemolytic activity of DP-AD**. **Figure S12.** Uptake efficiencies of DP-AD7 incubated at different temperature. **Figure S13.** Growth inhibitory effect of DP-AD7_anti-*acpP*_ in bacteria. **Figure S14.** Gene expression effect of DP-AD7_anti-*acpP*_ or DP-AD7_anti-*rpoD*_ in bacteria. **Figure S15.** Histological morphology of the organs. **Table S1.** The sequences, property and the number of His residues of DPPs. **Table S2.** The purity, *m/z* values and positive charge number of DPPs. **Table S3.** The solvents and concentration of store solutions of DPPs. **Table S4.** The size and zeta potential of DP-AD. **Table S5.** The positive ratio of bacteria after co-incubating with FAM-labeled DP-AD. **Table S6.** The minimum inhibitory concentration of DPPs to bacteria.

## Data Availability

All data generated or analysed during this study are included in this published article.
