# Supplementary material for: Amphipathic dendritic poly-peptides carrier to deliver antisense oligonucleotides against multi-drug resistant bacteria in vitro and in vivo
Source: J Nanobiotechnology. 2022 Apr 2;20:180. doi: 10.1186/s12951-022-01384-y (PMC8977034; doi:10.1186/s12951-022-01384-y)
Supplement: Supplementary file 1 — Additional file 1: Figure S1. The HPLC and mass spectra of DPP1-DPP6. Figure S2. The HPLC and mass spectra of DPP7-DPP12. Figure S3. The identification of L-DPP, ASOs, and DSPE-mPEG2000. Figure S4. The screening of the N/P ratio of ADs. Figure S5. The encapsulation of ASOs and binding rate of DSPE-mPEG2000 in the nanoparticles. Figure S6. Screening the molar ratio of DSPE-mPEG2000 in DP-AD. Figure S7. The characteristics of the nanoparticles. Figure S8. The serum stability of DP-AD. Figure S9. Uptake efficiencies and growth curves of DP-AD in E. coli and S. aureus. Figure S10. Uptake profiles of DP-AD by E. coli and S. aureus. Figure S11. The cytotoxicity and hemolytic activity of DP-AD. Figure S12. Uptake efficiencies of DP-AD7 incubated at different temperature. Figure S13. Growth inhibitory effect of DP-AD7anti-acpP in bacteria. Figure S14. Gene expression effect of DP-AD7anti-acpP or DP-AD7anti-rpoD in bacteria. Figure S15. Histological morphology of the organs. Table S1. The sequences, property and the number of His residues of DPPs. Table S2. The purity, m/z values and positive charge number of DPPs. Table S3. The solvents and concentration of store solutions of DPPs. Table S4. The size and zeta potential of DP-AD. Table S5. The positive ratio of bacteria after co-incubating with FAM-labeled DP-AD. Table S6. The minimum inhibitory concentration of DPPs to bacteria. [file 12951_2022_1384_MOESM1_ESM.docx]

**Title:** Amphipathic Dendritic Poly-peptides Carrier to Deliver Antisense Oligonucleotides against Multi-drug Resistant Bacteria *in vitro* and *in vivo*

*Zhou Chen, Xinggang Mao, Yue Hu, Mingzhi Wang, Bo Ma, Hui Zhao, Zheng Hou, Mingkai Li, Jingru Meng, Dan nie, Xiaoxing Luo, Xiaoyan Xue*

**Keywords:** Antibacterial strategy; Antisense; Dendritic poly-peptides; Multidrug-resistant bacteria; Nanoparticles


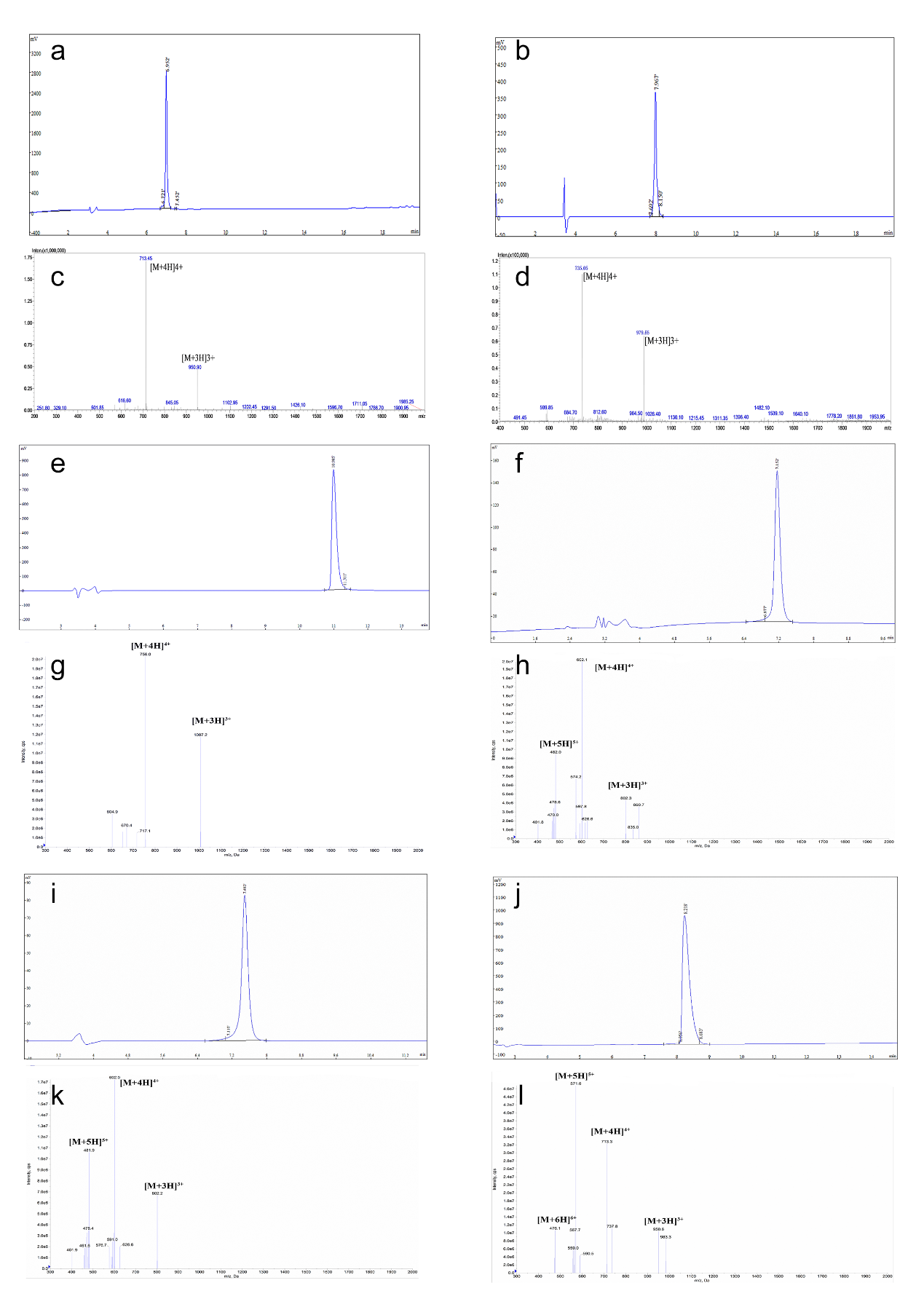


**Figure S1.** **The HPLC and mass spectra of DPP1-DPP6.** The purity of DPP1 was 98.19% (**a**), the [M+3]^3+^ *m/z* was 950.90 (**c**). The purity of DPP2 was 98.49% (**b**), the [M+3]^3+^ *m/z* was 979.65 (**d**). The purity of DPP3 was 99.34% (**e**), the [M+3]^3+^ *m/z* was 1007.2 (**g**). The purity of DPP4 was 98.06% (**f**), the [M+3]^3+^ *m/z* was 802.3 (**h**). The purity of DPP5 was 98.12% (**i**), the [M+3]^3+^ *m/z* was 802.2 (**k**). The purity of DPP6 was 98.18% (**j**), the [M+3]^3+^ *m/z* was 950.6 (**l**).


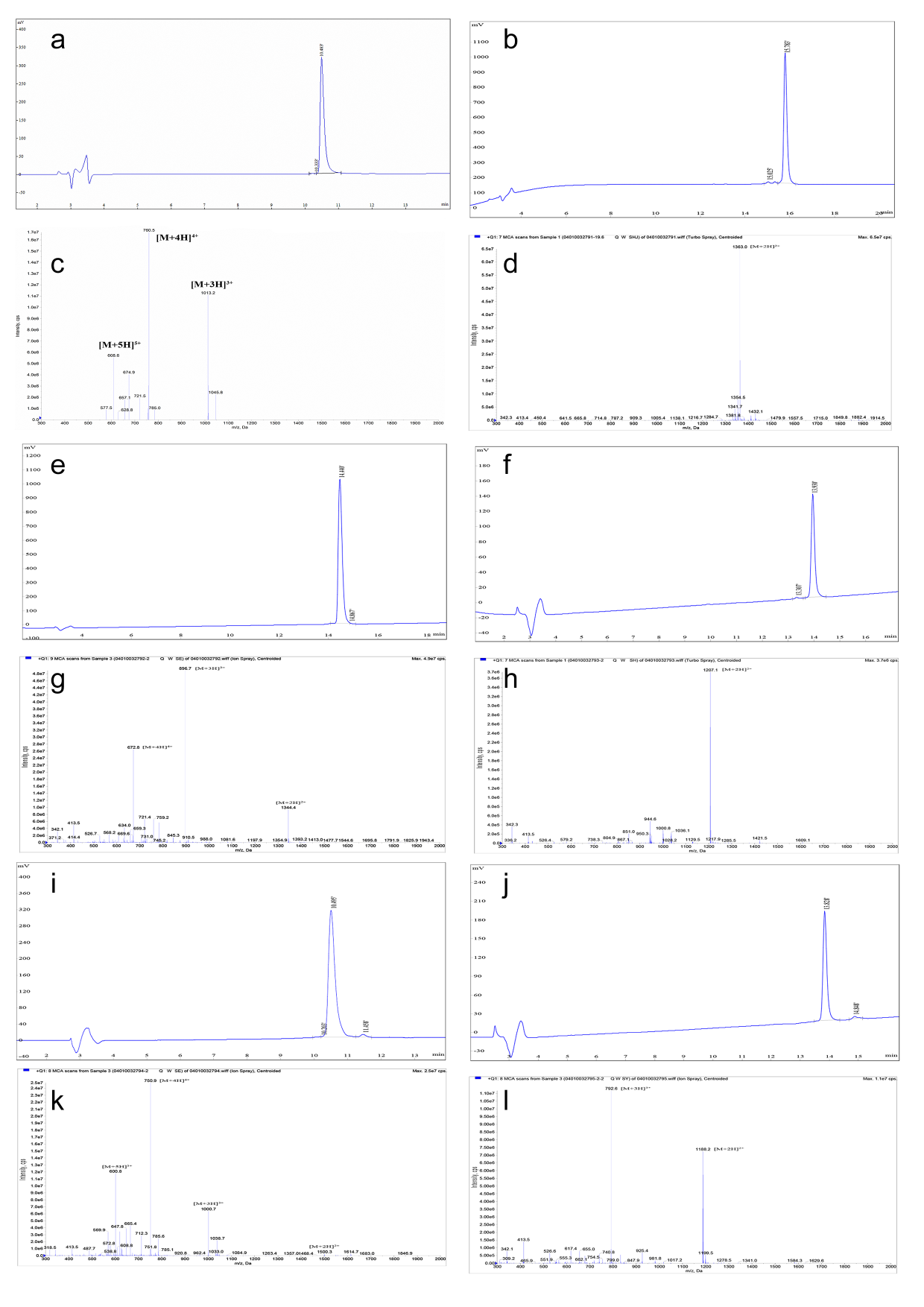


**Figure S2.** **The HPLC and mass spectra of DPP7-DPP12.** The purity of DPP7 was 98.06% (**a**), the [M+3]^3+^ *m/z* was 1013.2 (**c**). The purity of DPP8 was 98.09% (**b**), the [M+2]^2+^ *m/z* was 1363.0 (**d**). The purity of DPP9 was 99.82% (**e**), the [M+3]^3+^ *m/z* was 896.7 (**g**). The purity of DPP10 was 98.06% (**f**), the [M+2]^2+^ *m/z* was 1207.1 (**h**). The purity of DPP11 was 98.12% (**i**), the [M+3]^3+^ *m/z* was 1000.7 (**k**). The purity of DPP12 was 98.18% (**j**), the [M+2]^2+^ *m/z* was 1188.2 (**l**).


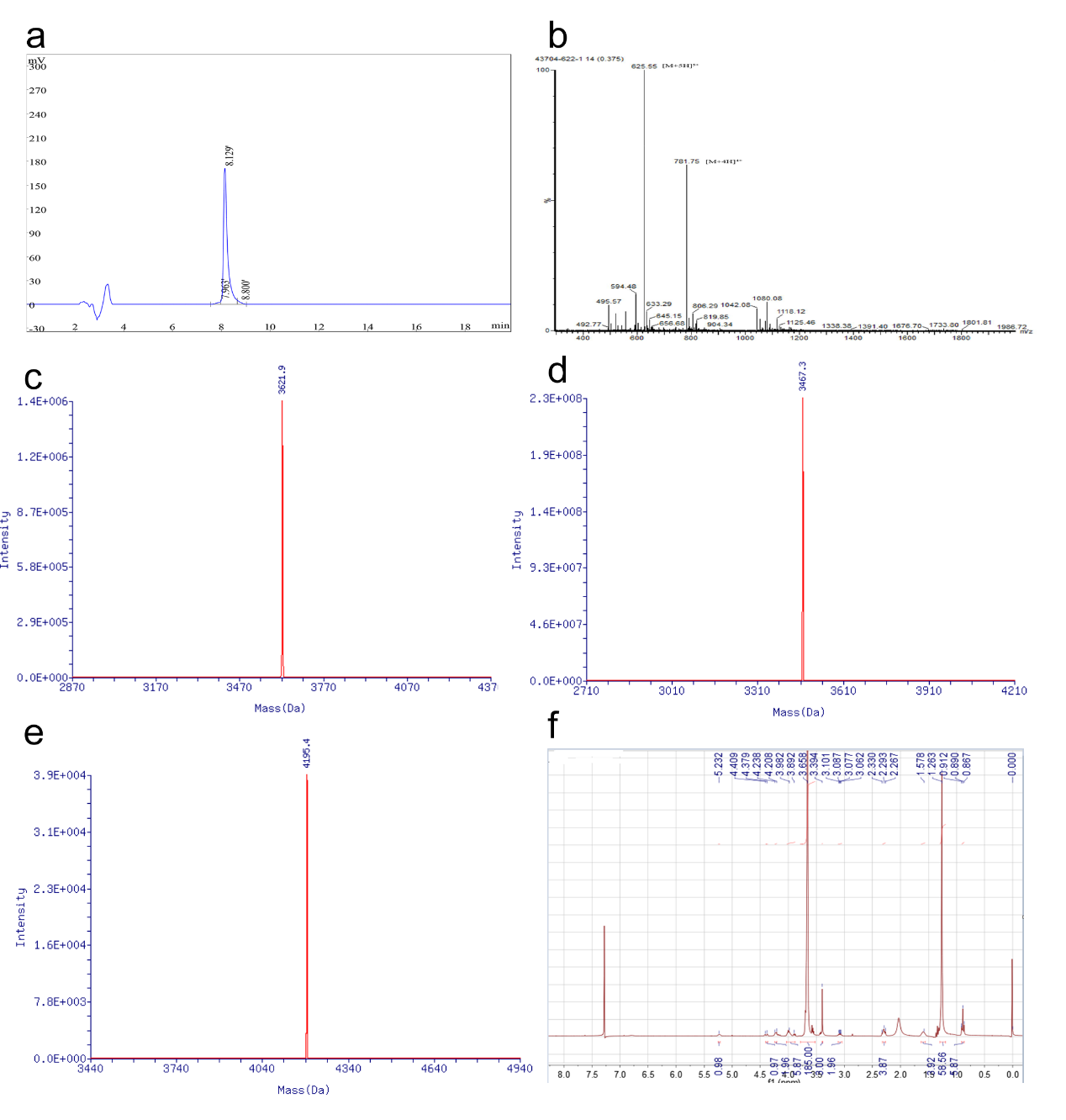


**Figure S3. The identification of L-DPP, ASOs, and DSPE-mPEG2000.** The HPLC and mass spectra of linear DPP (L-DPP). The purity of L-DPP was 96.12% (**a**), the [M+4]^4+^ *m/z* was 781.75 (**b**). The mass spectra of 2’-OMe modified ASOs (**c**), mismatched ASOs (**d**) and FAM-labled ASOs (**e**). The NMR spectrum of DSPE-mPEG2000 (**f**).


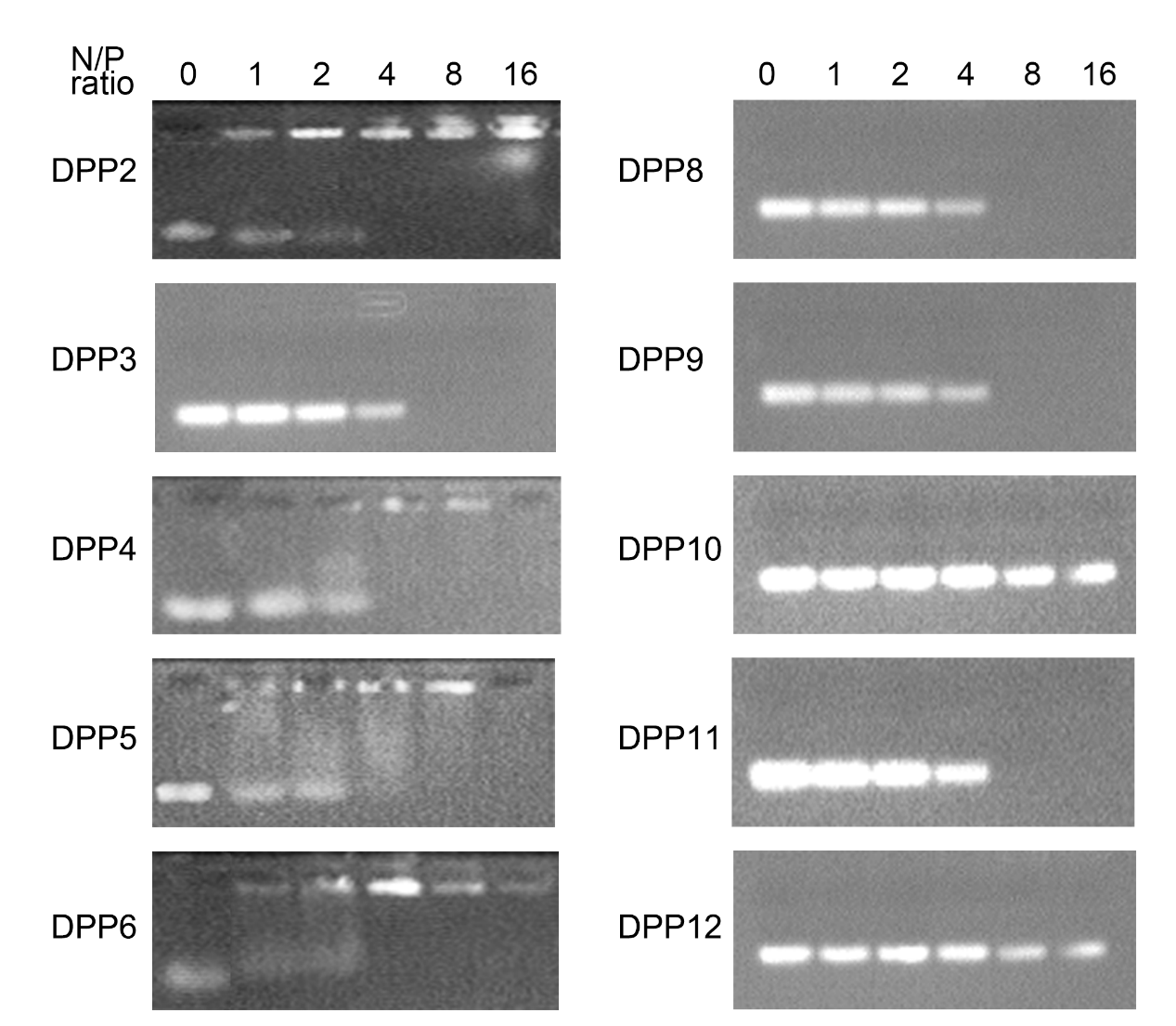


**Figure S4**. **The screening of the N/P ratio of ADs.** ADs with N/P molar ratio ranging from 1 to 16 of ASOs/DPP2, ASOs/DPP3, ASOs/DPP4, ASOs/DPP5, ASOs/DPP6, ASOs/DPP8, ASOs/DPP9, ASOs/DPP10, ASOs/DPP11, and ASOs/DPP12 by 1% agarose gel electrophoresis.


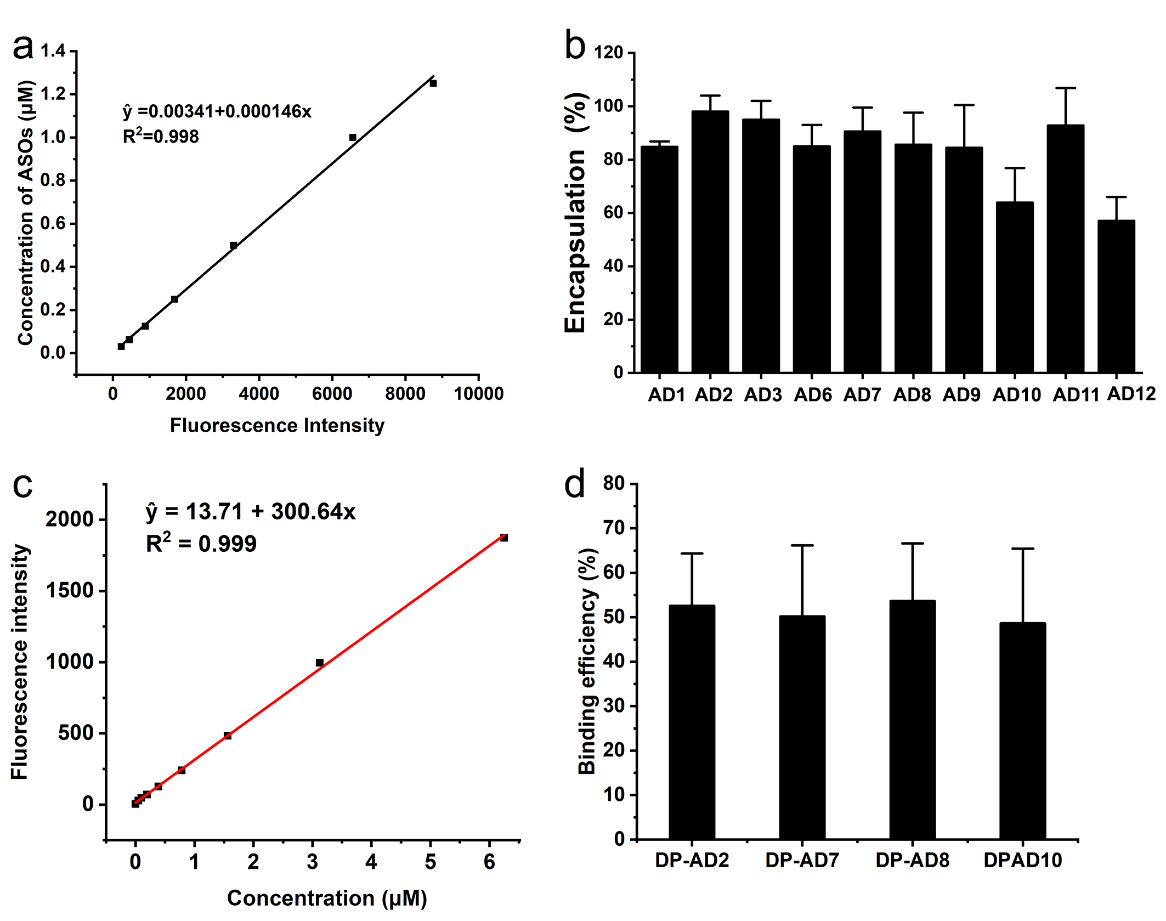


**Figure S5.** **The encapsulation of ASOs and binding rate of DSPE-mPEG2000 in the nanoparticles. a** Standard curve of FAM-labeled ASOs. Concentration versus fluorescence intensity. **b** The encapsulation of ASOs by different DPPs, the results showed that the encapsulation of ASOs was above 85% except DPP10 and DPP12. **c** Standard curve of FAM-labeled DSPE-mPEG2000. Fluorescence intensity versus the concentration of DSPE-mPEG2000. **d** The binding rate of DSPE-mPEG2000 in DP-AD2, DP-AD7, DP-AD8 and DP-AD10, the results showed that the binding rate of DPSE-mPEG2000 in DP-ADs were about 50%.


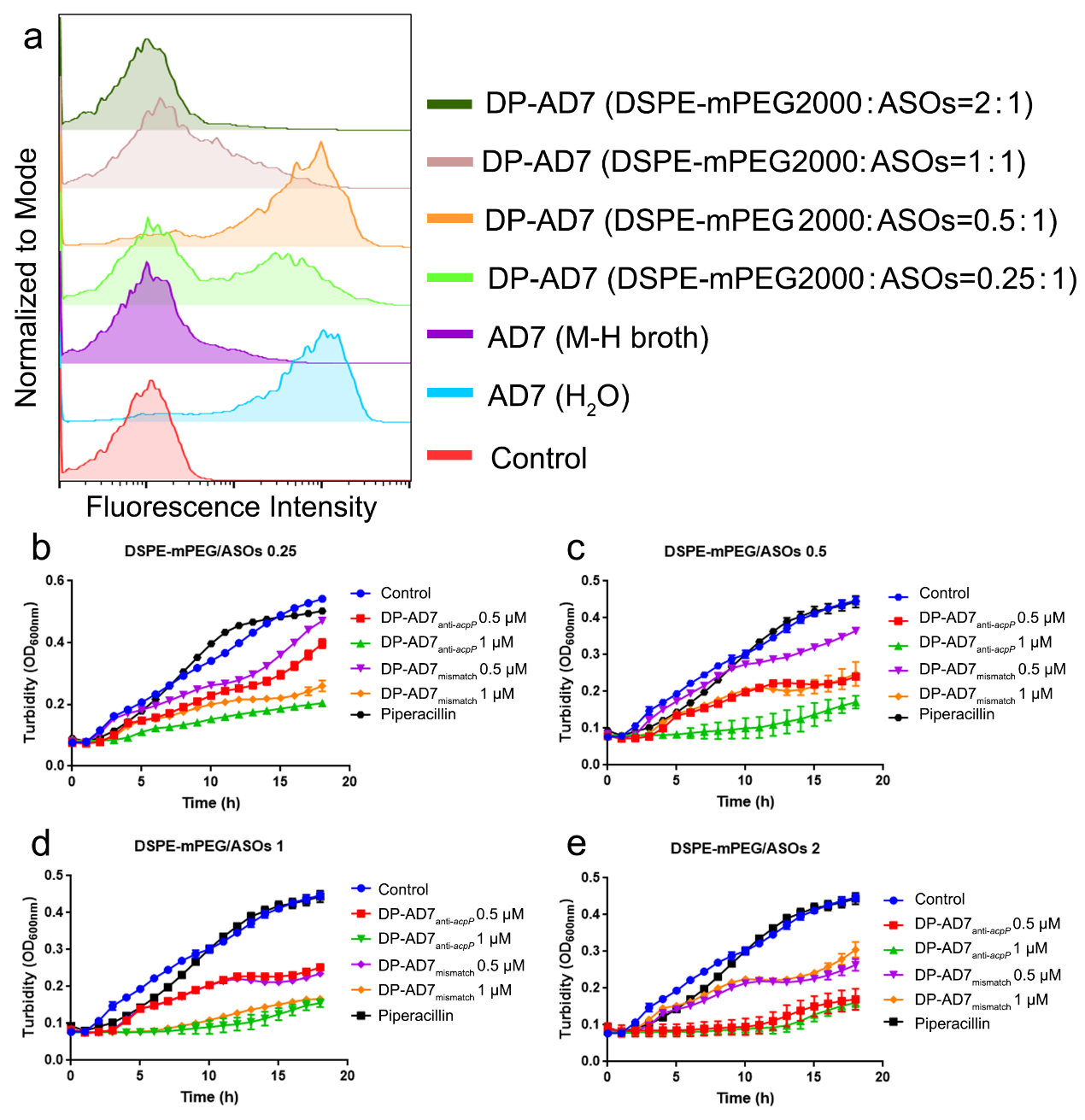


**Figure S6. Screening the molar ratio of DSPE-mPEG2000 in DP-ADs. a** FAM-positive ratio ESBLs-*E. coli* were detected by flow cytometry after incubating with DP-AD7_anti-_*_acpP_*, in which the DPSE-mPEG-PEG2000: ASOs rate ranged from 0.25 to 2, for 1 h in dark at 37 °C. AD7 in dd H_2_O and free ASOs were used as positive and negative control, respectively. There was the highest FAM positive rate and strongest fluorescent intensity when the rate of DSPE-mPEG2000: ASOs was 0.5. **b-e** The growth curve of ESBLs-*E. coli* treated with DP-AD7_anti-_*_acpP_* with DSPE-mPEG2000: ASOs of 0.25 (**b**), 0.5 (**c**), 1 (**d**) and 2 (**e**). Free ASOs and piperacillin were used as negative and positive control, respecitively. DP-AD7_anti-_*_acpP_* could significantly inhibit the bacterial growth compared with DP-AD7_mismatch_ groups when the molar rate was 0.25 or 0.5, but no significant difference when the molar rate was 1 or 2. To ensure the stability and the delivery efficiency, we chose the molar rate of DSPE-mPEG2000: ASOs = 0.5 to prepare DP-AD.


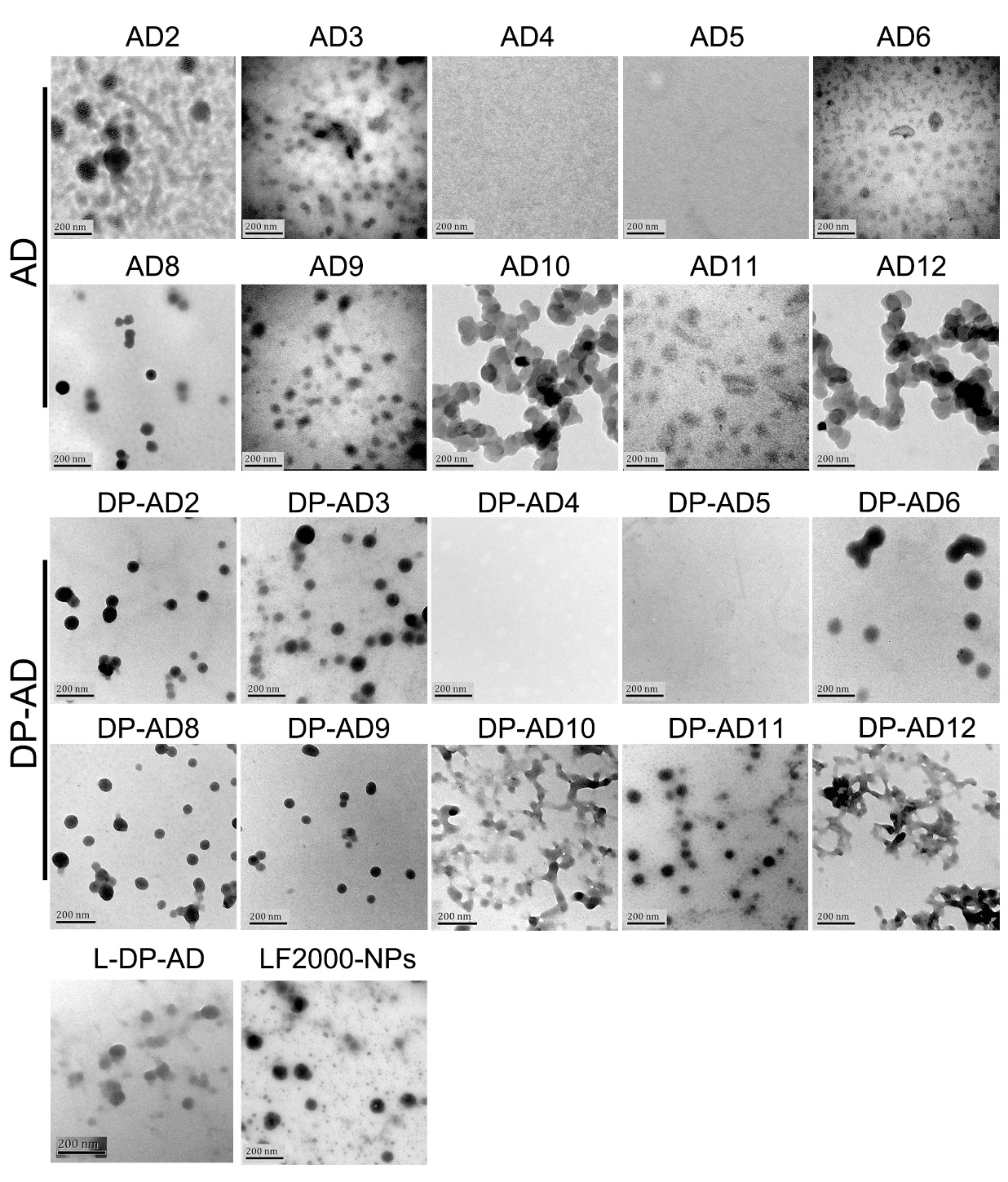


**Figure S7**. **The characteristics of the nanoparticles.** The morphologies of AD (upper panel), DP-AD (lower panel), L-DP-AD and LF2000-NPs observed by TEM.


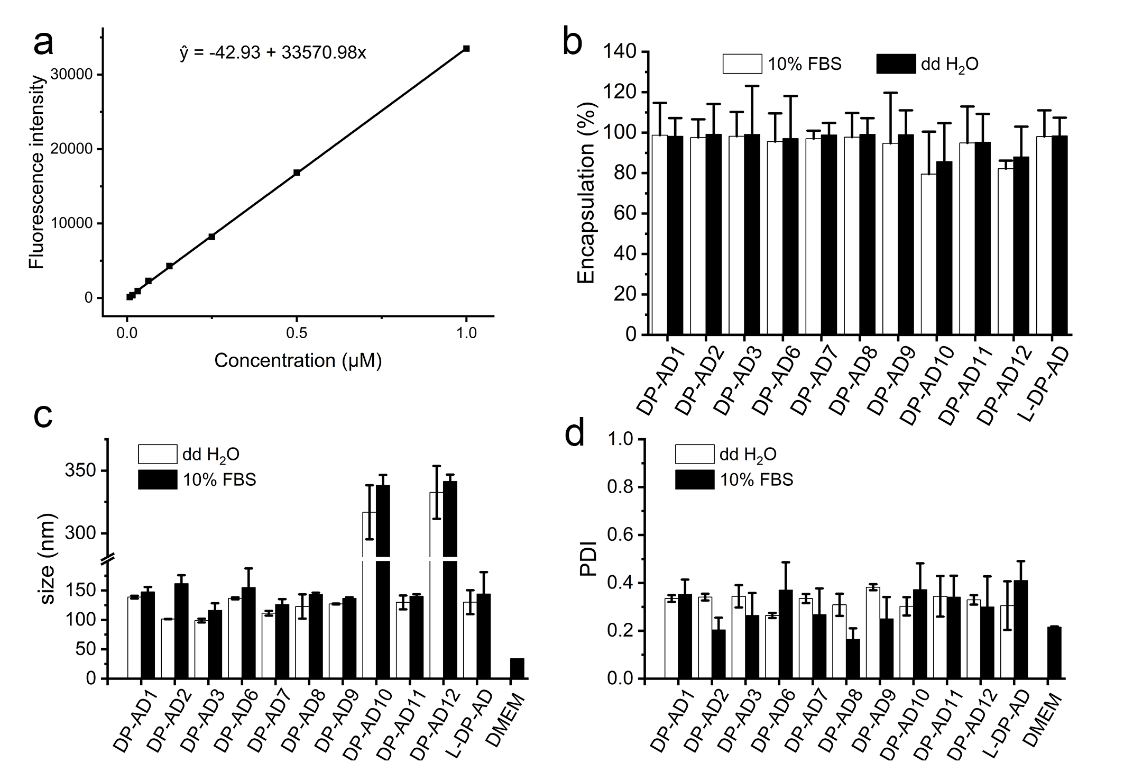


**Figure S8.** **The serum stability of DP-AD.** (**a**) Standard curve of FAM-labeled ASOs. Concentration versus fluorescence intensity. (**b**) The encapsulation rates of ASOs by DP-AD in water and diluted with equal volume of 10% FBS at 37 °C for 6 h. (**c**) The size of DP-AD in water and diluted with equal volume of 10% FBS. (**d**) The PDI of DP-AD in **c**.


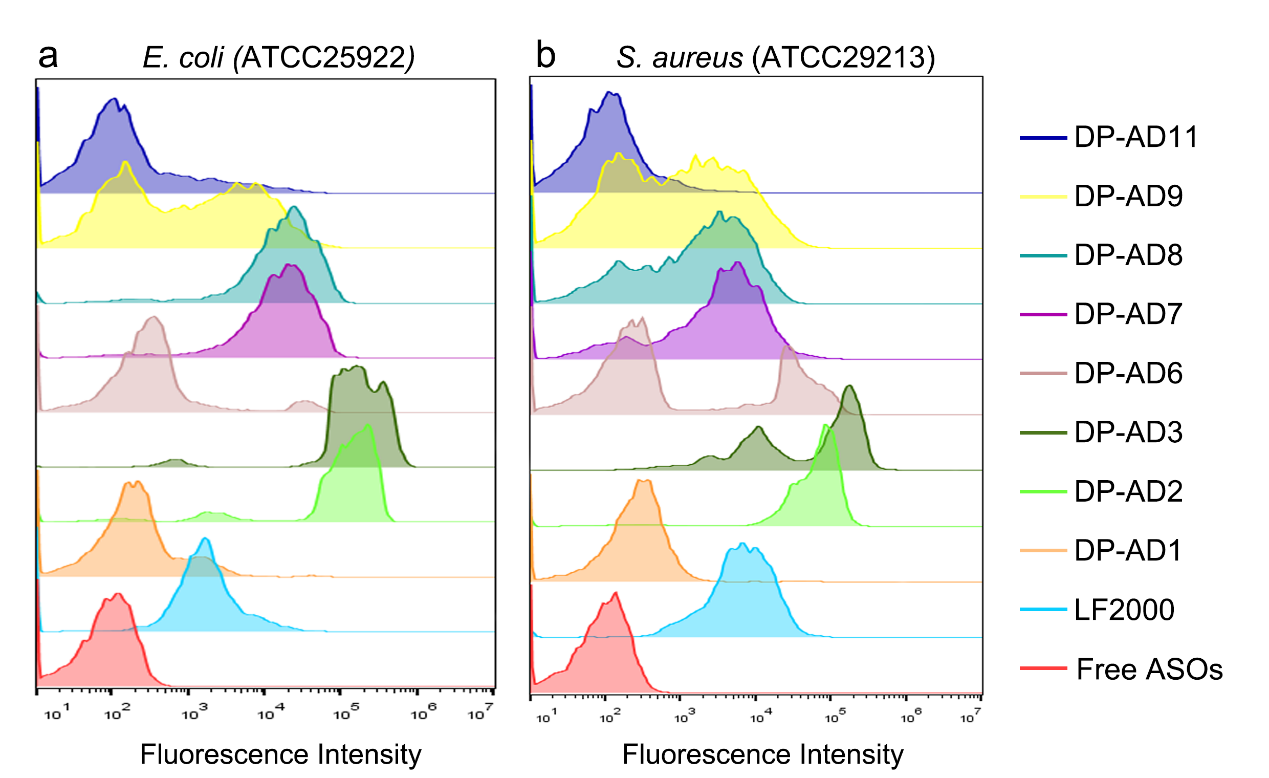


**Figure S9. Uptake efficiencies and growth curves of DP-AD in *E. coli* and *S. aureus*.** FAM-positive ratio of *E. coli* (**a**) and *S. aureus* (**b**) were tested by flow cytometry after incubation with FAM-labeled DP-ADs for 1 h in dark at 37 °C. Free FAM-labeled ASOs (red) and LF2000-NPs (light blue) were used as negative and positive controls, respectively.


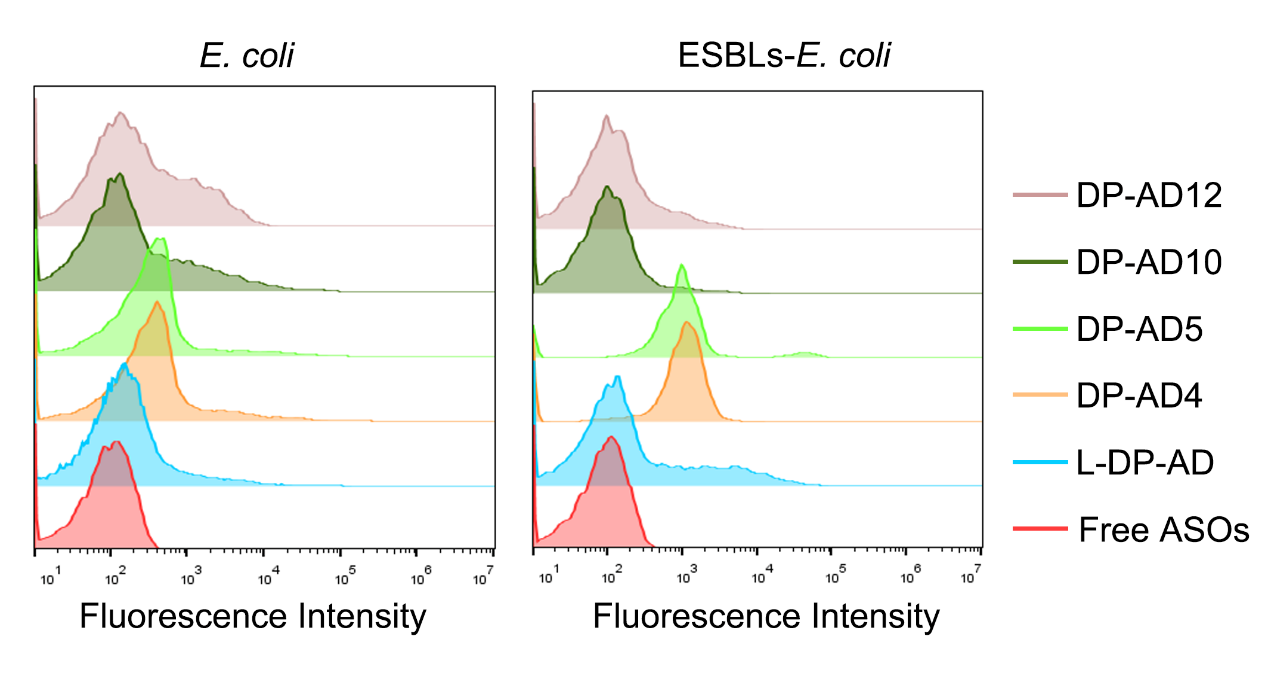


**Figure S10.** **Uptake profiles of DP-AD by *E. coli* and *S. aureus*.** FAM-positive ratio of *E. coli* (left panel) and ESBLs-*E. coli* (right panel) were tested by flow cytometry after incubating with FAM-labeled L-DP-AD (light blue), DP-AD4 (orange), DP-AD5 (light green), DP-AD10 (dark green) and DP-AD12 (light brown) for 1 h in dark at 37 °C, respectively. Free FAM-labeled ASOs (red) was used as negative control.


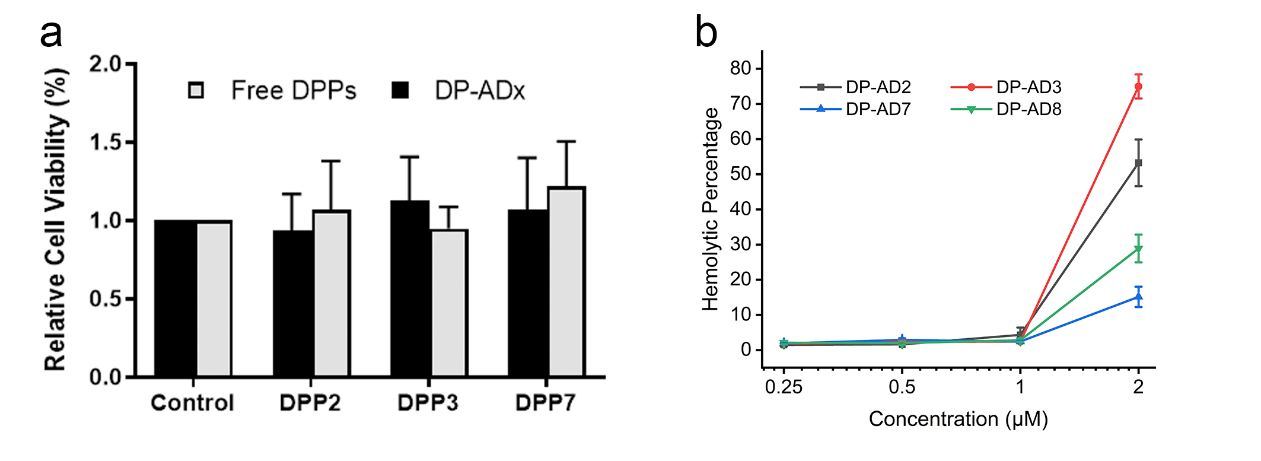


**Figure S11. The cytotoxicity and hemolytic activity of DP-AD**. **a** The cytotoxicity of 1 μM amphipathic DPP2, DPP3, DPP7 and DP-AD2, DP-AD3 and DP-AD7 in HIEC cells. **b** The hemolytic activity of DP-AD2, DP-AD3, DP-AD7 and DP-AD8 with the concentration range from 0.25 to 2 μM.


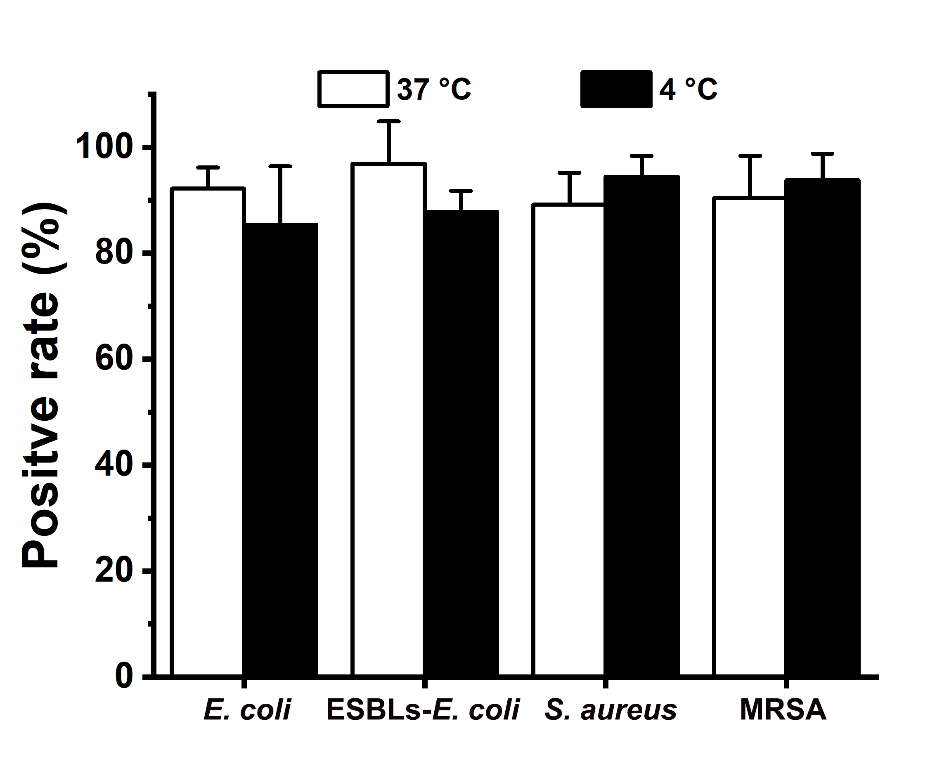


**Figure S12. Uptake efficiencies of DP-AD7 incubated at different temperature.** Uptake efficiency of DP-AD7 by the tested bacterial strains when incubated at 37 °C or 4 °C.


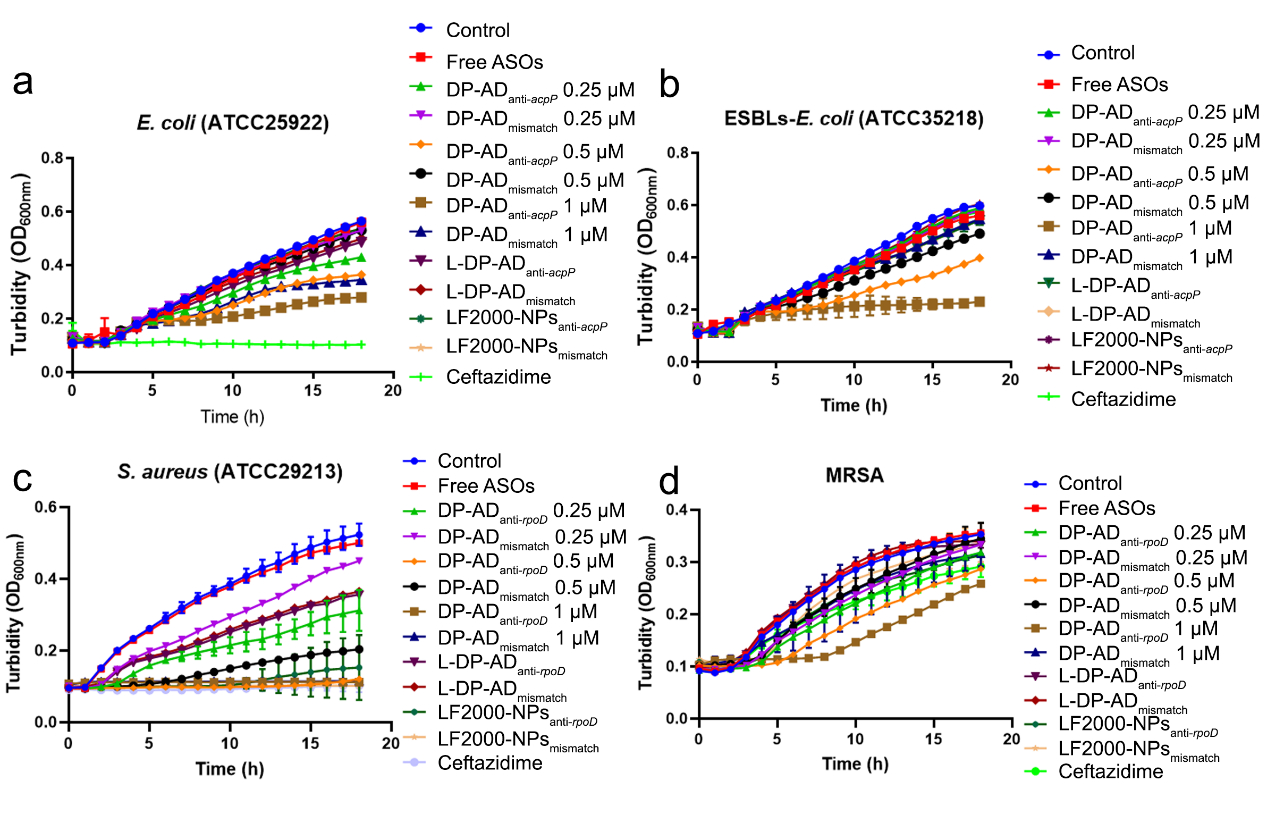


**Figure S13. Growth inhibitory effect of DP-AD7_anti-_*_acpP_* in bacteria.** Growth curves of *E. coli* (**a**), ESBLs-*E. coli* (**b**), *S. aureus* (**c**) and MRSA (**d**) treated with 0.25, 0.5 and 1 μM DP-AD7_anti-_*_acpP_* and DP-AD7_mismatch_, and L-DP-AD. LF2000-NPs and ceftazidime were used as positive control groups, while free ASOs were used as negative control.


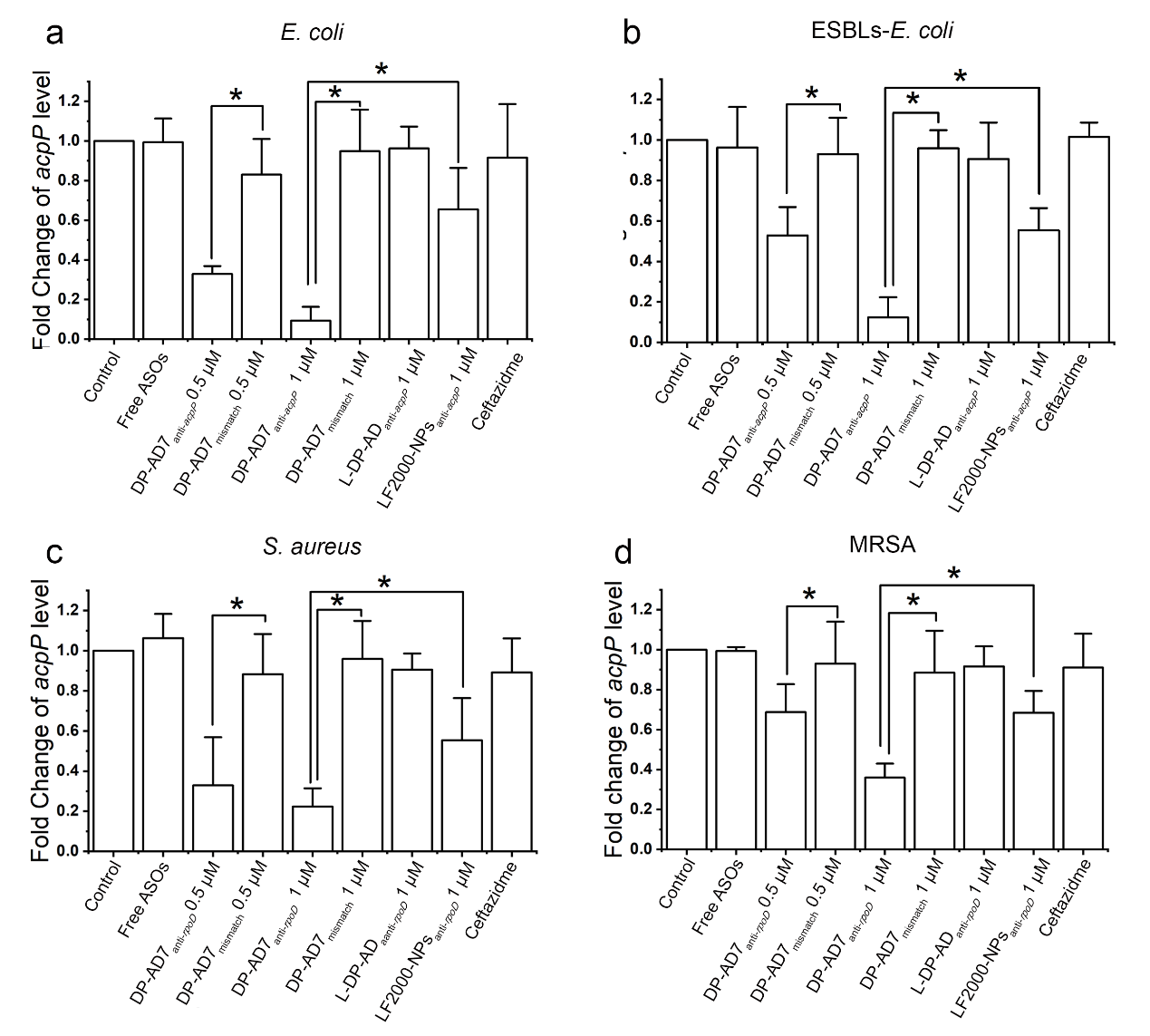


**Figure S14. Gene expression effect of DP-AD7_anti-_*_acpP_* or DP-AD7_anti-_*_rpoD_* in bacteria.** Fold change of *acpP* mRNA level of *E. coli* (**a**) and ESBLs-*E. coli* (**b**) or *rpoD* mRNA of *S. aureus* (**c**) and MRSA (**d**) treated with different concetrations of DP-AD7_anti-_*_acpP_* or DP-AD7_anti-_*_rpoD_*. Ceftazidime and LF2000-NPs were used as positive control, and free ASOs were used as negative control.


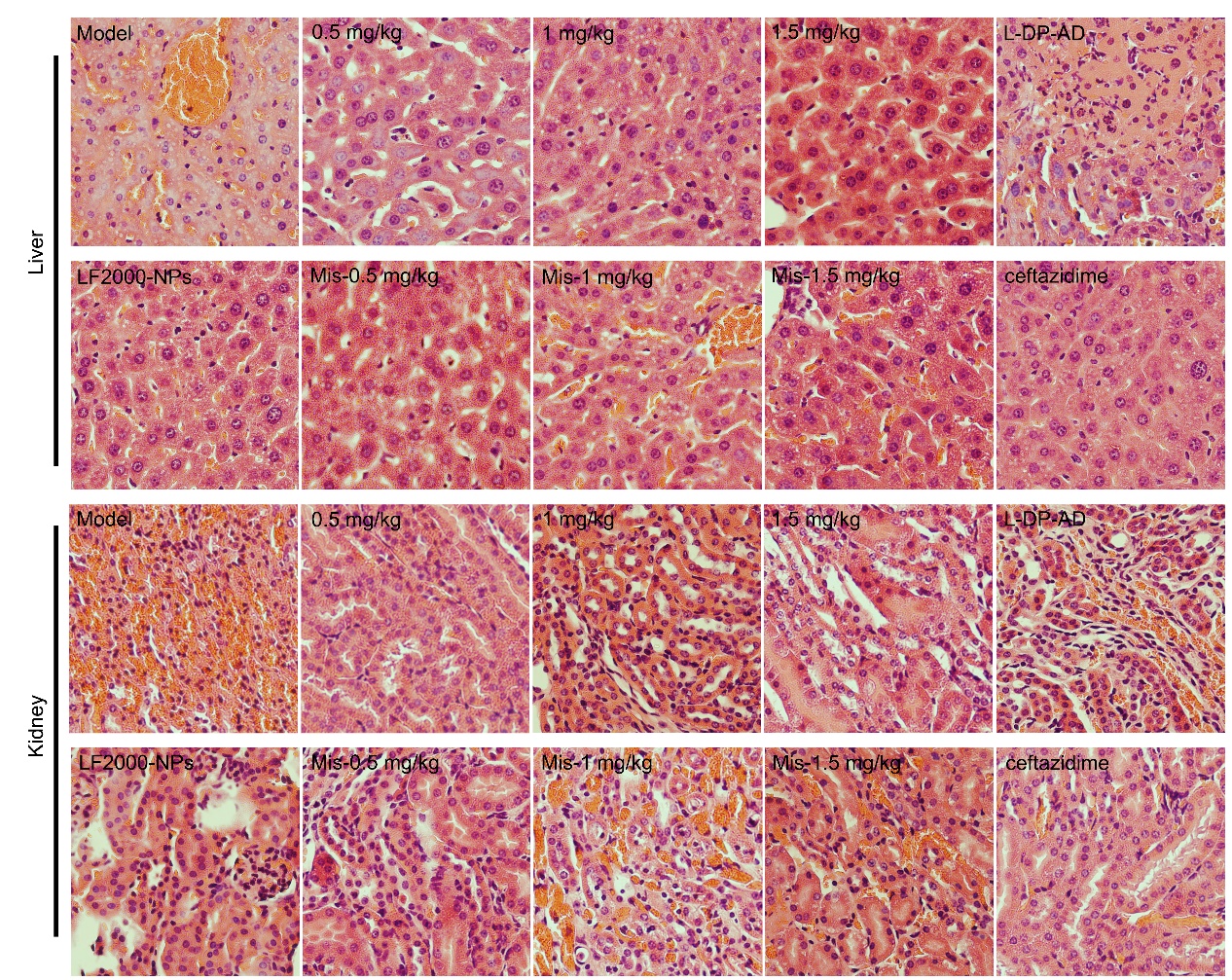


**Figure S15. Histological morphology of the organs.** Liver (upper panel) and kidney (lower panel) stained with hematoxylin and eosin in normal BALB/c mice and sepsis models treated with DP-AD7_anti-_*_acpP_* (0.5, 1 or 1.5 mg/kg), DP-AD7_mismatch_ (0.5, 1 or 0.5 mg/kg), L-DP-AD_anti-_*_acpP_*, LF2000-NPs_anti-_*_acpP_* and ceftazidime, respectively. (Original magnification, ×40).

**Supplementary Tables**

**Table S1.** The sequences, property and the number of His residues of DPPs.

| No. | Sequence ^a)^ | Property ^b)^ | Number of His residues |
| --- | --- | --- | --- |
| DPP1 | 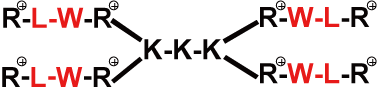 | H | 0 |
| DPP2 | 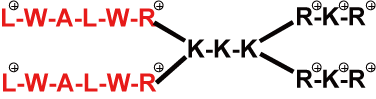 | A | 0 |
| DPP3 | 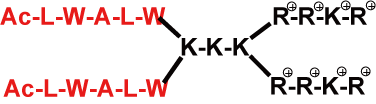 | A | 0 |
| DPP4 | 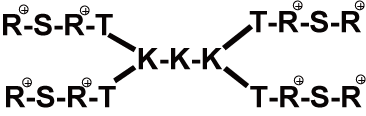 | H | 0 |
| DPP5 | 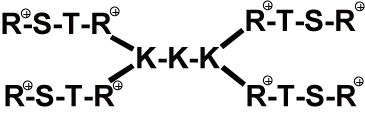 | H | 0 |
| DPP6 | 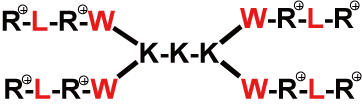 | H | 0 |
| DPP7 | 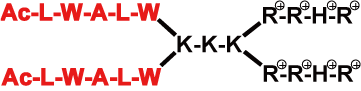 | A | 2 |
| DPP8 | 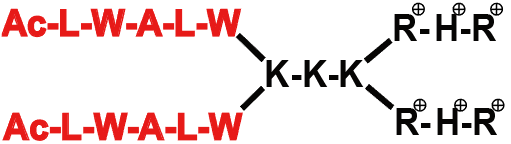 | A | 2 |
| DPP9 | 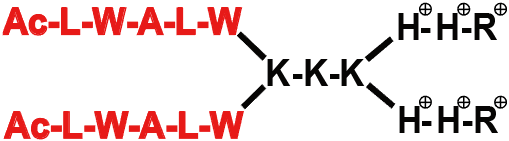 | A | 4 |
| DPP10 | 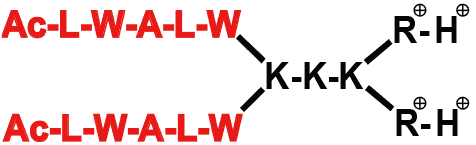 | A | 2 |
| DPP11 | 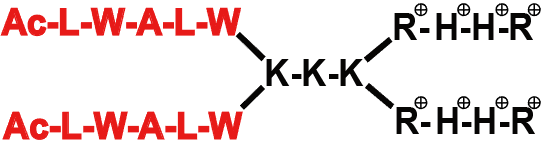 | A | 4 |
| DPP12 | 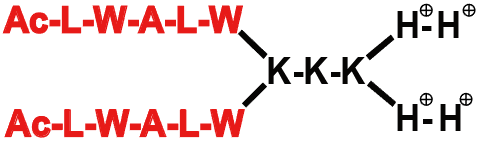 | A | 4 |
| L-DPP | Ac-LWALWK(Ac)K(Ac)K(Ac)RRHRRRHR^c)^ | | |

**Note:** ^a)^ The hydrophobic amino acids are presented in red, and the hydrophilic amino acids are presented in black. The positive charges are indicated by .

^b)^ “H” means hydrophilic, “A” means amphipathic.

^c)^ L-DPP was the amphipathic poly-peptides with the same amino acid of DPP7, 9 positive charge and 2 His residues.

**Table S2.** The purity, *m/z* values and positive charge number of DPPs.

| Peptide | Purity (%) | m/z | | Positive charge number |
| --- | --- | --- | --- | --- |
|  |  | Theoretical value | Measured value |  |
| DPP1 | 98.19 | 2849.7 | 2849.7 | 12 |
| DPP2 | 98.49 | 2935.7 | 2935.8 | 12 |
| DPP3 | 99.34 | 3018.7 | 3018.6 | 10 |
| DPP4 | 98.06 | 2403.8 | 2403.9 | 12 |
| DPP5 | 98.12 | 2403.8 | 2403.9 | 12 |
| DPP6 | 98.18 | 2848.6 | 2848.8 | 12 |
| DPP7 | 98.06 | 3036.9 | 3036.6 | 10 |
| DPP8 | 98.09 | 2724.3 | 2724.0 | 8 |
| DPP9 | 99.82 | 2686.3 | 2687.1 | 8 |
| DPP10 | 98.06 | 2411.9 | 2412.2 | 6 |
| DPP11 | 98.12 | 2998.5 | 2999.6 | 10 |
| DPP12 | 98.18 | 2373.4 | 2374.4 | 6 |
| L-DPP | 96.12 | 3121.6 | 3122.8 | 9 |

**Table S3.** The solvents and concentration of store solutions of DPPs.

| DPPs | Mass (mg) | Solvents | Volume (μl) | Concentration of store solutions (mM) |
| --- | --- | --- | --- | --- |
| DPP1 | 2.00 | dd H_2_O | 38.5 | 18.2 |
| DPP2 | 2.00 | Ethanol | 30.4 | 22.2 |
| DPP3 | 2.00 | 25% ACN | 33.0 | 20.0 |
| DPP4 | 2.00 | dd H_2_O | 49.8 | 16.7 |
| DPP5 | 2.00 | dd H_2_O | 49.8 | 16.7 |
| DPP6 | 2.00 | 20% ACN | 42.0 | 16.7 |
| DPP7 | 2.00 | 25% ACN | 33.0 | 20.0 |
| DPP8 | 2.00 | 25% ACN | 29.4 | 25.0 |
| DPP9 | 2.00 | 25% ACN | 29.8 | 25.0 |
| DPP10 | 2.00 | 25% ACN | 24.9 | 33.3 |
| DPP11 | 2.00 | 25% ACN | 33.3 | 20.0 |
| DPP12 | 2.00 | 25% ACN | 25.3 | 33.3 |
| L-DPP | 2.00 | 25% ACN | 28.5 | 22.5 |

**Table S4.** The size, polydispersity index (PDI) and zeta potential of DP-AD (*$\bar{x}$*± *s,* n=3).

| Nanoparticles | Size (nm) | | PDI | | Zeta potential (mV) |
| --- | --- | --- | --- | --- | --- |
|  | dd H_2_O | M-H broth | dd H_2_O | M-H broth |  |
| DP-AD1 | 150.4±17.4 | 155.4±8.6 | 0.161±0.09 | 0.207±0.06 | 33.05±0.07 |
| DP-AD2 | 135.9±8.8 | 141.5±2.1 | 0.213±0.07 | 0.188±0.03 | 31.15±2.76 |
| DP-AD3 | 131.2±23.3 | 131.8±14.6 | 0.162±0.06 | 0.276±0.02 | 32.55±5.87 |
| DP-AD6 | 138.1±7.6 | 134.6±9.2 | 0.104±0.02 | 0.160±0.01 | 31.40±11.17 |
| DP-AD7 | 134.1±31.0 | 132.5±29.0 | 0.326±0.04 | 0.300±0.02 | 31.40±1.27 |
| DP-AD8 | 131.2±21.1 | 147.3±14.7 | 0.212±0.05 | 0.218±0.01 | 31.51±1.32 |
| DP-AD9 | 140.3±14.8 | 150.6±12.4 | 0.234±0.11 | 0.149±0.02 | 27.92±2.13 |
| DP-AD10 | 495.5±120.1 | 700.3±85.4 | 0.357±0.14 | 0.362±0.12 | 8.942±0.32 |
| DP-AD11 | 135.6±9.6 | 149.7±9.6 | 0.125±0.08 | 0.155±0.08 | 25.44±3.21 |
| DP-AD12 | 378.2±86.9 | 680.6±75.4 | 0.386±0.18 | 0.401±0.15 | 10.61±2.55 |
| L-DP-AD | 123.1±7.6 | 130.5±12.7 | 0.416±0.21 | 0.415±0.13 | 30.20±1.80 |

**Table S5.** The positive ratio of bacteria after co-incubating with FAM-labeled DP-AD. (*$\bar{x}$*± *s,* n=3).

| Bacteria | *E. coli* (%) | ESBLs-*E. coli* (%) |
| --- | --- | --- |
| Free ASOs | 1.65±1.02 | 2.51±1.95 |
| LF2000 | 52.20±3.25 | 54.82±6.98 |
| DP-AD1 | 7.54±1.84 | 18.86±8.46 |
| DP-AD2 | 92.50±5.49 | 71.11±3.52 |
| DP-AD3 | 95.50±6.25 | 96.46±4.13 |
| DP-AD6 | 9.36±1.99 | 37.63±7.42 |
| DP-AD7 | 92.62±5.12 | 92.32±3.21 |
| DP-AD8 | 93.42±3.19 | 94.21±7.71 |
| DP-AD9 | 40.33±5.16 | 34.36±10.42 |
| DP-AD10 | 11.51±8.31 | 2.26±1.91 |
| DP-AD11 | 10.93±5.22 | 16.28±13.14 |
| DP-AD12 | 11.88±2.78 | 4.98±3.14 |

**Table S6.** The minimum inhibitory concentration of DPPs to bacteria (μg/ml).

|  | DPP2 | DPP3 | DPP7 | DPP8 |
| --- | --- | --- | --- | --- |
| *E. coli* | 16 | 16 | 16 | 32 |
| ESBLs-*E. coli* | 16 | 16 | 32 | 32 |
| *S. aureus* | 16 | 32 | 16 | 16 |
| MRSA | 16 | 16 | 32 | 16 |
